# New information on Late Triassic sauropodomorph dinosaurs provides support for the independent acquisition of postcranial skeletal pneumaticity in avemetatarsalian lineages

**DOI:** 10.1111/joa.70045

**Published:** 2025-09-25

**Authors:** Samantha L. Beeston, Daniela Schwarz, Paul Upchurch, Paul M. Barrett, Patrick Asbach, Philip D. Mannion

**Affiliations:** ^1^ Department of Earth Sciences University College London London UK; ^2^ Museum für Naturkunde, Leibniz Institute for Research on Evolution and Biodiversity Berlin Germany; ^3^ Fossil Reptiles, Amphibians, and Birds Section Natural History Museum London UK; ^4^ Department of Radiology Charité—Universitätsmedizin Berlin, Corporate Member of Freie Universität Berlin and Humboldt‐Universität Zu Berlin Berlin Germany

**Keywords:** computed tomography, evolution, postcranial skeletal pneumaticity, Sauropoda, Sauropodomorpha, Triassic

## Abstract

Within Avemetatarsalia, postcranial skeletal pneumaticity (PSP) occurs in pterosaurs, as well as theropod (including extant Aves) and sauropod dinosaurs. However, the evolutionary origins of PSP in the latter clade remain largely unknown, with few studies assessing species closely related to, but outside, the sauropod radiation, that is, early‐branching sauropodomorphs. Furthermore, most proposed identifications of PSP in early‐branching sauropodomorphs relate to external indicators of internal pneumaticity, for example, the presence of vertebral subfossae. To address this deficit, we CT scanned representative elements from the vertebral columns of the early‐branching sauropodomorphs *Thecodontosaurus antiquus*, *Pantydraco caducus*, *Ruehleia bedheimensis* and *Plateosaurus longiceps*, all from the Late Triassic of Europe. These new data were compared with the small number of early‐branching sauropodomorphs with published vertebral CT scan data, namely the Late Triassic Brazilian species, *Buriolestes schultzi*, *Pampadromaeus barberenai* and *Macrocollum itaquii*. Based on the sampled vertebrae, PSP is absent in *Buriolestes*, *Pampadromaeus*, *Pantydraco* and probably *Thecodontosaurus*. It is possible that the neural arches of the posterior cervical vertebrae of *Thecodontosaurus* possess PSP, but this can only be interpreted from broken transverse cross‐sections and not CT scans. The posterior cervical vertebrae of *Ruehleia* possess PSP in the neural arches; however, their corresponding centra, along with the centra and neural arches of the anterior–middle dorsal vertebrae, are apneumatic. *Plateosaurus* possesses PSP in the neural arches of the middle cervical vertebrae through to the middle dorsal vertebrae, whereas the presacral centra are apneumatic. Where present, pneumatic internal chambers are neither exclusively camerate nor camellate, nor do they align with the ‘protocamerate’ bone structure previously described in the posterior cervical and anterior dorsal vertebrae of *Macrocollum*. From external indicators, PSP might be present in the sacral neural arches of *Ruehleia* and *Plateosaurus* but is absent in the caudal vertebrae. However, our results reveal that PSP cannot be unambiguously determined from external indicators; subfossae do not always communicate with internal chambers; and internal chambers sometimes communicate with undivided fossae. PSP in early‐branching sauropodomorphs probably evolved first in the neural arches of the posterior cervical vertebrae, expanding anteriorly and posteriorly along the vertebral column. Furthermore, the distribution of PSP in Late Triassic early‐branching sauropodomorphs does not appear to be correlated with body size. Finally, our results lend support to the idea that pterosauromorphs, theropods and sauropodomorphs evolved PSP in the Late Triassic independently of each other.

## Graphical abstract

PSP in early‐branching sauropodomorphs probably evolved first in the neural arches of the posterior cervical vertebrae, expanding anteriorly and posteriorly along the vertebral column. The distribution of PSP in Late Triassic early‐branching sauropodomorphs does not appear to be correlated with body size. Our results lend support to the idea that pterosauromorphs, theropods and sauropodomorphs evolved PSP in the Late Triassic independently of each other.

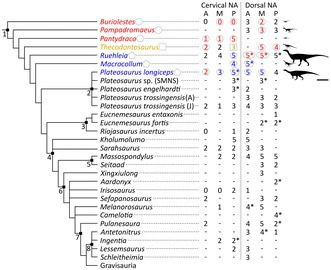

## 1 INTRODUCTION

Sauropods were gigantic, quadrupedal dinosaurs (Upchurch et al., 2004), with the largest species, such as *Patagotitan mayorum*, attaining masses exceeding 40 tonnes (Benson et al., 2014; Carballido et al., 2017; Klein et al., 2011; Sander et al., 2011). The ancestors of sauropods, early‐branching members of Sauropodomorpha, were much smaller, with estimated body masses of 8.5 kg and 1.1 tonne in the Late Triassic species *Pampadromaeus barberenai* and *Ruehleia bedheimensis*, respectively (Benson et al., 2014; McPhee et al., 2018). At the non‐sauropod sauropodomorph–true sauropod transition, some species had already attained much larger sizes, exemplified by the Early Jurassic sauropod *Ledumahadi mafube*, which weighed 12 tonnes (McPhee et al., 2018). It has been hypothesised that, to facilitate the evolution of long necks and large body size, sauropodomorphs evolved postcranial skeletal pneumaticity (PSP) within elements along the axial skeleton (Britt, 1997; Wedel, 2007, 2009), with this being well‐developed and extensive in neosauropods and closely related lineages (e.g., Cerda et al., 2012; Schwarz et al., 2007; Wedel, 2003a, 2003b).

PSP occurs via a respiratory air sac system wherein air sacs branch into smaller pneumatic diverticula, which are lined with pneumatic bone structure (Müller, 1908). The latter is able to resolve (i.e., pneumatise) bone, thereby invading skeletal elements and replacing the spongious part of the bone with air. Pneumatisation of a bone occurs via the communication between external pneumatic fossae (to which the diverticula are adhered) and internal pneumatic chambers. In addition to sauropodomorphs, PSP has been recorded in two further lineages of avemetatarsalian archosaurs, pterosauromorphs and theropods, with the latter including extant Aves (Aureliano et al., 2025; Benson et al., 2012; Britt, 1993, 1997; Butler et al., 2009, 2012; Müller, 1908; O'Connor, 2006; Wedel, 2003a, 2006, 2007, 2009). The evolution of PSP in extinct avemetatarsalians has been linked with the development of a respiratory air sac system homologous in form and function to that of extant Aves (Britt, 1993; Wedel, 2003a, 2007, 2009; Wedel et al., 2000; Witmer, 1997).

Whereas the origin and distribution of PSP in pterosauromorphs and theropods has been well studied, it remains less clear in sauropodomorphs (Aureliano et al., 2022, 2025; Aureliano, Almeida, et al., 2024; Aureliano, Ghilardi, et al., 2024; Benson et al., 2012; Britt, 1993; Butler et al., 2009; O'Connor & Claessens, 2005). Within early‐branching sauropodomorphs, PSP has been interpreted as present in the axial columns of several taxa that possess foramina within fossae (or subdivided fossae) on the neural arches. However, these purported occurrences of PSP are primarily based on external indicators of internal pneumaticity. PSP can only be unambiguously assessed from natural breaks in bone, destructive sectioning, traditional x‐rays or CT/synchrotron scanning. Currently, the best ways to accurately assess the presence of PSP are the latter three methods, which can directly image any communication between external pneumatic fossae and internal pneumatic chambers. In the absence of cross‐sectional internal imaging data confirming whether external fossae communicate internally, one might misidentify the true pneumatic characteristics of early‐branching sauropodomorphs.

To date, the only early‐branching sauropodomorphs with published CT scan data on the axial skeleton are *Buriolestes schultzi* (Aureliano et al., 2022), *Pampadromaeus barberenai* (Aureliano et al., 2022) and *Macrocollum itaquii* (Aureliano, Ghilardi, et al., 2024), all from the Late Triassic of Brazil. Of these taxa, *Macrocollum* is the only one known to possess PSP. Therefore, to gain deeper insight into the origin and early evolution of PSP in Sauropodomorpha, anatomical evidence from additional early members of the clade needs to be evaluated. Here, we present external and CT scan‐reconstructed internal anatomical data of representative elements from the axial columns of the Late Triassic European early‐branching sauropodomorphs *Thecodontosaurus antiquus*, *Pantydraco caducus*, *Ruehleia bedheimensis* and *Plateosaurus longiceps*. We combine these data with published information on contemporaneous sauropodomorph taxa from elsewhere to provide an expanded view of the distribution and early evolution of postcranial pneumaticity in early sauropodomorphs.

### 1.1 Institutional abbreviations

AMNH, American Museum of Natural History, New York, USA; BRSMG, Bristol Museum and Art Gallery, Bristol, United Kingdom; BRSUG, University of Bristol Geology Department, Bristol, United Kingdom; MB.R., Museum für Naturkunde, collection of fossil Reptilia, Berlin, Germany; MfN, Museum für Naturkunde, Berlin, Germany; MSF, Sauriermuseum Frick, Frick, Switzerland; NHMUK, Natural History Museum, London, United Kingdom; SMNS, Staatliches Museum für Naturkunde, Stuttgart, Germany.

### 1.2 Anatomical abbreviations

The terminology used to describe the vertebral laminae and fossae follows Wilson (1999) and Wilson et al. (2011), respectively. Abbreviations: ACDL, anterior centrodiapophyseal lamina; Ca, caudal vertebra; CDF, centrodiapophyseal fossa; Ce, cervical vertebra; D, dorsal vertebra; LPF, lateral pneumatic foramen/fossa; PACPRF, parapophyseal centroprezygapophyseal fossa; PCDL, posterior centrodiapophyseal lamina; POCDF, postzygapophyseal centrodiapophyseal fossa; PRCDF, prezygapophyseal centrodiapophyseal fossa; PRPADF, prezygapophyseal parapodiapophyseal fossa; PSP, postcranial skeletal pneumaticity; S, sacral vertebra; SPOF, spinopostzygapophyseal fossa; SPRF, spinoprezygapophyseal fossa.

## 2 MATERIALS AND METHODS

### 2.1 Material examined

#### 2.1.1 *Thecodontosaurus antiquus* Morris, 1843

*Thecodontosaurus antiquus* is one of the earliest dinosaurs to have been named (Morris, 1843; Riley & Stutchbury, 1840) and the first to be erected from the Triassic (Benton, 2012). Material attributed to the *Thecodontosaurus* hypodigm has been found at two Late Triassic (Rhaetian) localities in Bristol, United Kingdom: Durdham Down and Tytherington (Ballell et al., 2020; Benton et al., 2000). Herein, we describe material from the Durdham Down locality (Table 1).

**TABLE 1:** Summary of material examined herein.

Taxon	Cervical vertebrae	Cervical ribs	Dorsal vertebrae	Dorsal ribs	Sacral vertebrae	Caudal vertebrae
*Thecodontosaurus antiquus* (BRSMG)	Cb 4151–4152; Cb 4155; Cb 4167	–	Cb 4153–4154; Cb 4221; C 4533; Cb 4714	Ca 7466; Cb 4169–4170; Cb 4174; Cb 4255–4256; Cb 4528; Cb 4285	–	Cb 4164; Cb 4714
*Pantydraco caducus* (NHMUK)	PV RUP24	–	–	–	–	–
*Ruehleia bedheimensis* (MB.R)	4718.20; 4718.50–57	4718.112	4718.41–49; 4718.67–72	4718.1–19; 4718.21–25	4718.27; 4774; 6751	4718.28–38; 4718.60–66; 4718.113
*Plateosaurus longiceps* (MB.R)	4404.16–24; 4430.13–21	4430.96; 4430.98–104; 4430.181; 4430.205	4404.25–36; 4430.22–36	4405.10–11; 4430.106–111; 4430.119–130; 4430.206–207	4405.12–13; 4405.85; 4430.37; 4392; 4398.25	4405.15–50; 4430.39–80; 4430.209

#### 2.1.2 *Pantydraco caducus* Yates, 2003a

The holotype specimen of *Pantydraco caducus*, NHMUK PV RUP24, was discovered in a Late Triassic (Rhaetian) quarry in Pantyffynnon, south Wales, United Kingdom, and initially referred to *Thecodontosaurus* sp. by Kermack (1984). Subsequently, the material was designated as a new species of *Thecodontosaurus*, *T*. *caducus* (Yates, 2003a), before being assigned as a distinct genus, with the new combination, *Pantydraco caducus* (Galton et al., 2007). *Pantydraco* and *Thecodontosaurus* have been consistently recovered as closely related taxa and represent some of the earliest branching sauropodomorphs (Beccari et al., 2021; Müller et al., 2018; Peyre de Fabrègues & Allain, 2020; Schaeffer, 2024).

#### 2.1.3 *Ruehleia bedheimensis* Galton, 2001a

The holotype and referred specimen of *Ruehleia bedheimensis* (Table 1) derive from the Late Triassic (Norian) and were discovered and excavated at Großer Gleichberg, near Römhild, Germany, by Hugo Rühle von Lilienstern in 1932–1933, who initially referred the material to *Plateosaurus plieningeri*, providing only a brief description (Rühle von Lilienstern et al., 1952; Werneburg, 1999a, 1999b). Galton (2001a, 2001b) subsequently erected *Ruehleia bedheimensis* for these remains. He provided a brief diagnosis of the species and stated that the material would be redescribed elsewhere. Unfortunately, this has yet to come to fruition, meaning that *Ruehleia* has never received a detailed published description. In phylogenetic analysis, *Ruehleia* is generally recovered as an early‐branching sauropodomorph that is the sister taxon to Plateosauridae (Peyre de Fabrègues & Allain, 2020; Rauhut et al., 2020; Schaeffer, 2024).

#### 2.1.4 *Plateosaurus longiceps* Jaekel, 1913

The validity of the numerous named *Plateosaurus* species has been discussed at length (see Beccari et al., 2021; Galton, 2001a, 2001b; Nau et al., 2020; Regalado Fernández et al., 2023; Regalado Fernández & Werneburg, 2022; Schaeffer, 2024; Yates, 2003b, 2010). Material of *Plateosaurus* from the Late Triassic (Norian) clay pit locality in Halberstadt, Sachsen‐Anhalt, Germany, reposited in the MfN collection (Table 1), has been described by Jaekel (1914), Galton (1985), and Bonaparte (1999). Although uncertainties remain with regard to the taxonomic assignment of the Halberstadt material (see also Galton & Kermack, 2010), these specimens are typically referred to as *Plateosaurus longiceps* by most authors (see Regalado Fernández et al., 2023), which we follow here.

### 2.2 Methods

Two blocks containing dorsal and caudal vertebrae of *Thecodontosaurus* (BRSMG Cb 4164 and BRSMG Cb 4714) were CT scanned at the University of Bristol in November 2024. Due to the high density of both blocks, the CT scans of *Thecodontosaurus* have a low contrast resolution, and the distinction between rock and bone is difficult to determine. Ce1–Ce5 of *Pantydraco caducus* were CT scanned at the NHMUK in September 2018. Ce6–Ce9 were subsequently CT scanned at the same facility in January 2025, although at lower contrast resolution. Two cervical and two dorsal vertebrae of *Ruehleia* (Ce9, MB.R.4718.56; Ce10, MB.R.4718.57; D1, MB.R.4718.41; D5, MB.R.4718.45) were CT scanned at the Department of Radiology at the Charité in Berlin, Germany, in November 2024. Remains from two Halberstadt *Plateosaurus* specimens, MB.R.4404 (‘Skeleton 25’, note that Bonaparte (1999) erroneously reported this as MB.R.2090) and MB.R.4430 (‘Skeleton C’), were scanned at the same facility as *Ruehleia*. Three cervical and four dorsal vertebrae were scanned from MB.R.4404 (Ce4, MB.R.4404.18; Ce7, MB.R.4404.21; Ce9, MB.R.4404.23; D1, MB.R.4404.25; D3, MB.R.4404.27; D6, MB.R.4404.30; D10, MB.R.4404.34), and two cervical and three dorsal vertebrae were scanned from MB.R.4430 (Ce9, MB.R.4430.20; Ce10, MB.R.4430.21; D2, MB.R.4430.23; D4, MB.R.4430.25; D9, MB.R.4430.30) in November 2024 and February 2025, respectively.

The material of *Thecodontosaurus* was scanned on a Nikon XTH 225ST X‐ray tomography scanner with a tube voltage of 180 kV and a voxel size of 0.63 mm for BRSMG Cb4164 and a tube voltage of 162 kV with a voxel size of 0.10 mm for BRSMG Cb4714. The cervical vertebrae of *Pantydraco* were scanned on a Nikon Metrology HMX ST 225 micro‐CT scanner with a tube voltage of 215 kV and a voxel size of 0.127 mm for Ce1–Ce5, and a tube voltage of 220 kV with a voxel size of 0.058 mm for Ce6–Ce9. *Plateosaurus* and *Ruehleia* were CT scanned by using a 320‐section multidetector CT unit (Aquillion Prime; Canon Medical Systems, Otawara, Japan). A helical scan mode with a rotation time of 1.0 s was chosen. The tube voltage was set to 135 kV and the voxel size varied between 0.2 and 0.7 mm.

CT scans were analysed, and screenshots of CT slices taken in Avizo 9.7 (FEI Visualization Science Group; https://www.thermofisher.com) and 3D Slicer 5.8.0 (https://www.slicer.org/). The cervical vertebrae of *Pantydraco* were surface scanned using an Artec Spider handheld scanner (Artec 3D, Santa Clara, CA, USA; https://www.artec3d.com/portable‐3d‐scanners/artec‐spider), and the subsequent three‐dimensional mesh was aligned and created in Artec Studio 16 Professional (www.artec3d.com/3d‐software/artec‐studio). Figures were assembled, outlined and annotated in Inkscape 1.4 (https://inkscape.org/), and colour density maps were generated in ParaView 5.13.2 (https://www.paraview.org/).

### 2.3 Assessment of pneumaticity criteria

Issues associated with defining ‘pneumatic correlates’, in particular concerning vertebral fossae, have been discussed at length (e.g., Benson et al., 2012; Britt, 1993; O'Connor, 2006; Wedel, 2003a, 2005, 2007; Yates et al., 2012). Britt (1993) established five diagnostic criteria for recognising pneumatic skeletal features in modern birds (and, by extension, in fossil species (Wedel, 2005)): large external foramina; external fossae with crenulate texture; bones with thin outer walls; smooth or crenulate tracks (grooves); and internal chambers with foramina. Yates et al. (2012) included an additional diagnostic criterion to this list: the position of a potential pneumatic structure on the bone surface (i.e., homologous consistency of position). Based on their study on archosaur PSP, O'Connor (2006) concluded that the only reliable indicators for pneumaticity are cortical foramina or communicating fossae that connect to large internal chambers (see also Benson et al., 2012), that is, fossae with subfossae (Apaldetti et al., 2018). Based on comparisons with extant birds, Wedel (2007) concluded that fossae can only be interpreted as pneumatic if they contain sharp‐lipped foramina that communicate with internal cavities, or if they are heavily sculpted and are divided into numerous subfossae. Here, we follow O'Connor (2006) and Wedel (2007) in regarding an element as pneumatic only if it possesses direct communication between external fossae and internal chambers and/or they are divided into subfossae. The pneumatic terminology used throughout this contribution follows the definitions outlined in Table 2.

**TABLE 2:** Summary of pneumatic terms used herein.

Term	Definition
Foramen	Anatomical passage, aperture (Britt, 1993). Any hole or opening passing through cortical bone (O'Connor, 2006).
Fossa	Surficial depression (Britt, 1993). Any concavity positioned in an anatomical surface (O'Connor, 2006).
Nutrient foramen	Vascular foramen/foramina with nutrient vessels supplying the medullary tissues interior of vertebral centra (O'Connor, 2006).
Pneumatic fossa	Excavations, typically conical in form (Britt, 1993). Excavations that are broad in contour and are not enclosed by osteal margins to form a foramen (Wedel, 2003b).
Protocamerate	Pneumatic tissue with properties of both camellae and camerae: structures which are not large enough to be considered camerae, but also present a camellate array internally (Aureliano, Ghilardi, et al., 2024).
Pseudo‐polycamerate	Chaotic apneumatic trabecular chambers infilled with blood and fat tissues but resemble the fractals of the pneumatic polycamerate (Aureliano et al., 2022).
Spongiosa/Cancellous	Spongy or cancellous bone wherein the volume of pore space is higher than the volume of bone tissue. Depending on the degree of porosity, spongiosa is subdivided into fine cancellous bone, coarse cancellous bone and trabecular bone, from lower to higher porosity (Francillon‐Vieillot et al., 1989). Solid, apneumatic (Wedel, 2005).
Trabecular bone	A type of cancellous bone in which trabeculae show a precise three‐dimensional spatial arrangement which reflects mechanical forces acting on the bone (Francillon‐Vieillot et al., 1989). Rod‐shaped bone tissue in cancellous bone that provides lightweight internal support (Woodruff et al., 2017).
True lateral pneumatic foramen (pleurocoel)	Depressions in the lateral walls of vertebrae with at least partially sharply outlined borders that cut deeply into the outer wall of the vertebra (Janensch, 1947).

## 3 RESULTS

Below, we describe skeletal structures potentially relevant to pneumaticity in the axial columns of *Thecodontosaurus*, *Pantydraco*, *Ruehleia* and *Plateosaurus*. These features are determined from a combination of first‐hand observations of external (and where possible, internal) anatomy, coupled with the interpretation of CT scan data.

### 3.1 Thecodontosaurus antiquus

Most of the *Thecodontosaurus* vertebrae examined are incomplete and preserved in blocks of matrix. This means that, in general, only one surface of each element is exposed and available for the evaluation of external features. As such, the following descriptions are based on the best‐preserved elements only. The serial positions of each element have been determined based on comparisons with referred material of *Thecodontosaurus* (Ballell et al., 2020), as well as the vertebral columns of *Pantydraco* (SLB, personal observation) and *Buriolestes schultzi* (Müller et al., 2018).

#### 3.1.1 Cervical vertebrae

**FIGURE 1:**
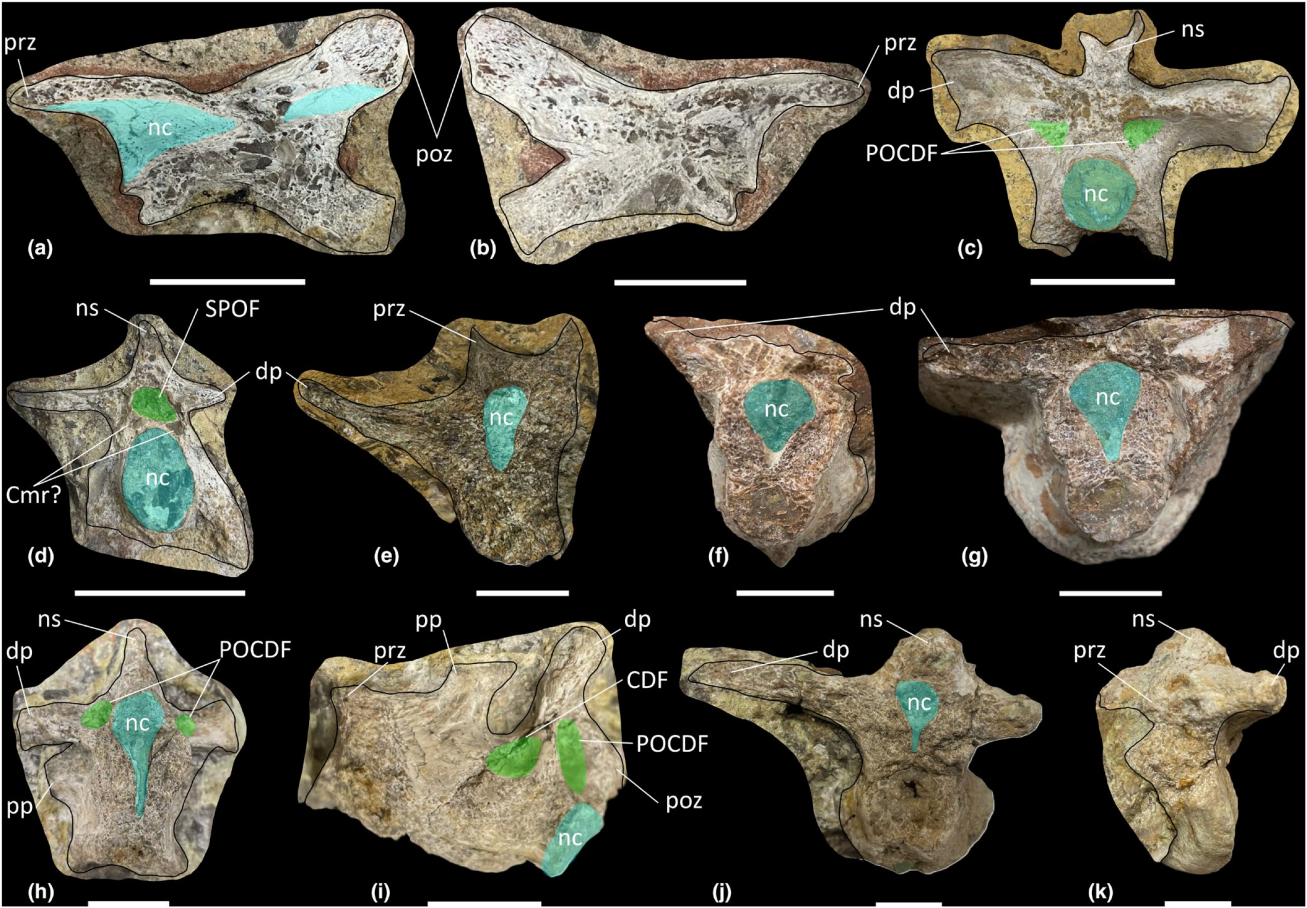
Presacral vertebrae of *Thecodontosaurus antiquus*. (a, b) Anterior cervical vertebra parasagittal cross‐section: (a) Cb 4151 right half and (b) Cb 4152 left half; (c, d) posterior cervical vertebrae transverse cross‐section: (c) Cb 4155 and (d) Cb 4167; (e–k) middle–posterior dorsal vertebrae: (e) Cb 4221 transverse cross‐section, (f) Cb 4154 transverse cross‐section and (g) Cb 4154 transverse cross‐section; (h, i) Cb 4153: (h) transverse cross‐section and (i) parasagittal cross‐section; (j, k) C 4533: (j) transverse cross‐section and (k) anterolateral view mirrored. CDF, centrodiapophyseal fossa; Cmr, camerae; dp, diapophysis; nc neural canal; ns, neural spine; POCDF, postzygapophyseal centrodiapophyseal fossa; poz, postzygapophysis; pp., parapophysis; prz, prezygapophysis; SPOF, spinopostzygapophyseal fossa. Key: Blue highlight, neural canal; green highlight, fossa. Scale bars = 2 cm.

The *Thecodontosaurus* hypodigm includes one anterior (BRSMG Cb 4151–Cb 4152; Figure 1a,b) and two posterior cervical vertebrae (BRSMG Cb 4155 and Cb 4167; Figure 1c,d). The anterior cervical vertebra is preserved as two lateral halves on separate blocks that have been broken along the parasagittal midline of the vertebra, and some internal bone has been lost from both sides. Matrix from corresponding areas on each block is the same lithology and colour, confirming this is one element that has been split apart. The posterior cervical vertebrae are incomplete and transversely broken along the posterior half of each element.

Lateral surfaces of the cervical centra are inaccessible due to incomplete preservation or the relevant surface being obscured by matrix. Broken surfaces of both the anterior and posterior cervical vertebrae reveal a spongious internal texture (Figure 1a–d). In the anterior cervical vertebra, the trabeculae are most densely packed close to the posterior cotyle and postzygapophyses, whereas there are some less densely packed trabeculae towards the middle of the centrum on both halves of the vertebra (Figure 1a,b). The anterior cervical vertebra appears to lack neural arch fossae, whereas the exposed surfaces of the posterior cervical vertebrae reveal the presence of postzygapophyseal centrodiapophyseal fossae (POCDF) and a spinopostzygapophyseal fossa (SPOF) (Figure 1c,d). However, the presence or absence of other neural arch fossae cannot be determined from the exposed surfaces of the posterior cervical vertebrae. Close to where the POCDF and SPOF are positioned, the neural arches of the posterior cervical vertebrae possess less‐densely packed trabeculae than in their centra. In BRSMG Cb 4155, small cavities occur dorsomedial to each POCDF (Figure 1c), and in BRSMG Cb 4167, small cavities occur all around the SPOF (Figure 1d). In particular, there appear to be two larger cavities located just ventrolateral to the SPOF in BRSMG Cb 4167.

#### 3.1.2 Dorsal vertebrae

**FIGURE 2:**
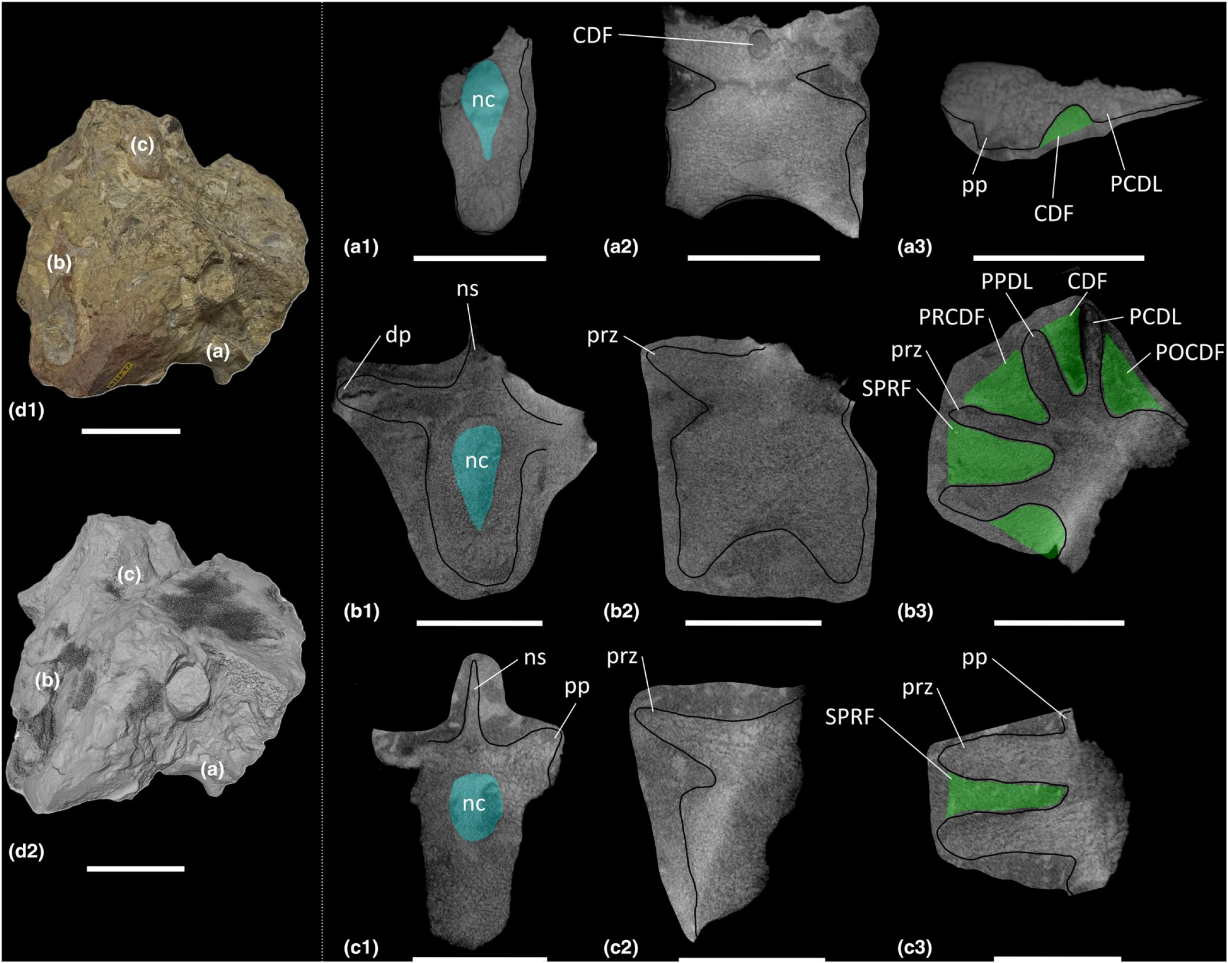
CT scan data of *Thecodontosaurus antiquus* dorsal vertebrae in Cb 4714 block. (a) element A, (b) element B, (c) element C; (a1–c1) transverse cross‐section, (a2–c2) parasagittal cross‐section, (a3–c3) horizontal cross‐section; (d1) photograph of block, (d2) surface scan of block. CDF, centrodiapophyseal fossa; dp, diapophysis; nc, neural canal; ns, neural spine; PCDL, posterior centrodiapophyseal lamina; POCDF, postzygapophyseal centrodiapophyseal fossa; pp., parapophysis; PPDL, parapodiapophyseal lamina; PRCDF, prezygapophyseal centrodiapophyseal fossa; prz, prezygapophysis; SPRF, spinoprezygapophyseal fossa. Key: Blue highlight, neural canal; green highlight, fossa. Scale bars = 2 cm (a–c), 5 cm (d).

The block BRSMG Cb 4714 preserves three dorsal vertebrae (Figure 2), and an additional five dorsal vertebrae are sufficiently preserved to be included in this description (BRSMG Cb 4221, Figure 1e; BRSMG Cb 4154, Figure 1f,g [two vertebrae in separate blocks]; BRSMG Cb 4153, Figure 1h,i; and BRSMG C 4533, Figure 1j,k). All dorsal vertebrae are identified as middle–posterior elements owing to the position of the parapophyses and diapophyses on the neural arches. Due to the preservation of all eight dorsal vertebral elements and the low contrast resolution of the CT scans, more specific positions cannot be determined. Other than the three dorsal vertebrae in block BRSMG Cb 4714, all dorsal vertebrae are broken along differing transverse sections, and their lateral surfaces are inaccessible due to incomplete preservation. Therefore, the presence or absence of the lateral depression described for the BRSUG *Thecodontosaurus* dorsal vertebrae by Ballell et al. (2020) cannot be determined in the BRSMG specimens. Of the BRSMG dorsal vertebrae, Cb 4153 is the best preserved and possesses a centrodiapophyseal fossa (CDF) and POCDF (Figure 1h,i). A prezygapophyseal centrodiapophyseal fossa (PRCDF) is clearly absent and, as such, BRSMG Cb 4153 is probably a dorsal vertebra located more posterior to D7, given that the PRCDF is lost in the posterior dorsal vertebrae of most early‐diverging sauropodomorphs, including *Ruehleia* and *Plateosaurus* (see below). The presence or absence of the spinoprezygapophyseal fossa (SPRF) and SPOF cannot be determined in the BRSMG dorsal vertebrae, owing to incomplete preservation. However, one of the CT scanned dorsal vertebrae (element B of BRSMG Cb 4714) possesses a PRCDF, CDF, POCDF and SPRF (Figure 2b3), but the posterior half of the neural arch of this element is not preserved, meaning that the presence or absence of the SPOF cannot be ascertained.

Inspection of the dorsal vertebrae reveals a spongious internal texture in the centra and neural arches. The centra of BRSMG Cb 4154 (both elements), BRSMG Cb 4533 and element A of BRSMG Cb 4714 possess large cavities with less densely packed trabeculae than other dorsal centra, but these cavities do not appear to communicate with any external fossae (Figures 1f,g,j and 2a1,a2).

#### 3.1.3 Dorsal ribs

Eight dorsal ribs are preserved, comprising two partial ribs with proximal ends (BRSMG Cb 4528 and Cb 4285) and six rib shafts (BRSMG Ca 7466; Cb 4169–Cb 4170; Cb 4174; and Cb 4255–Cb 4256). The posterior surface of BRSMG Cb 4528 is exposed, and it possesses a gentle concavity between the capitulum and tuberculum. Within this concavity, there is a small nutrient foramen. However, BRSMG Cb 4285 lacks an equivalent concavity or foramen. Broken surfaces of all dorsal ribs reveal a solid internal texture at both the proximal ends and along the shaft.

#### 3.1.4 Caudal vertebrae

**FIGURE 3:**
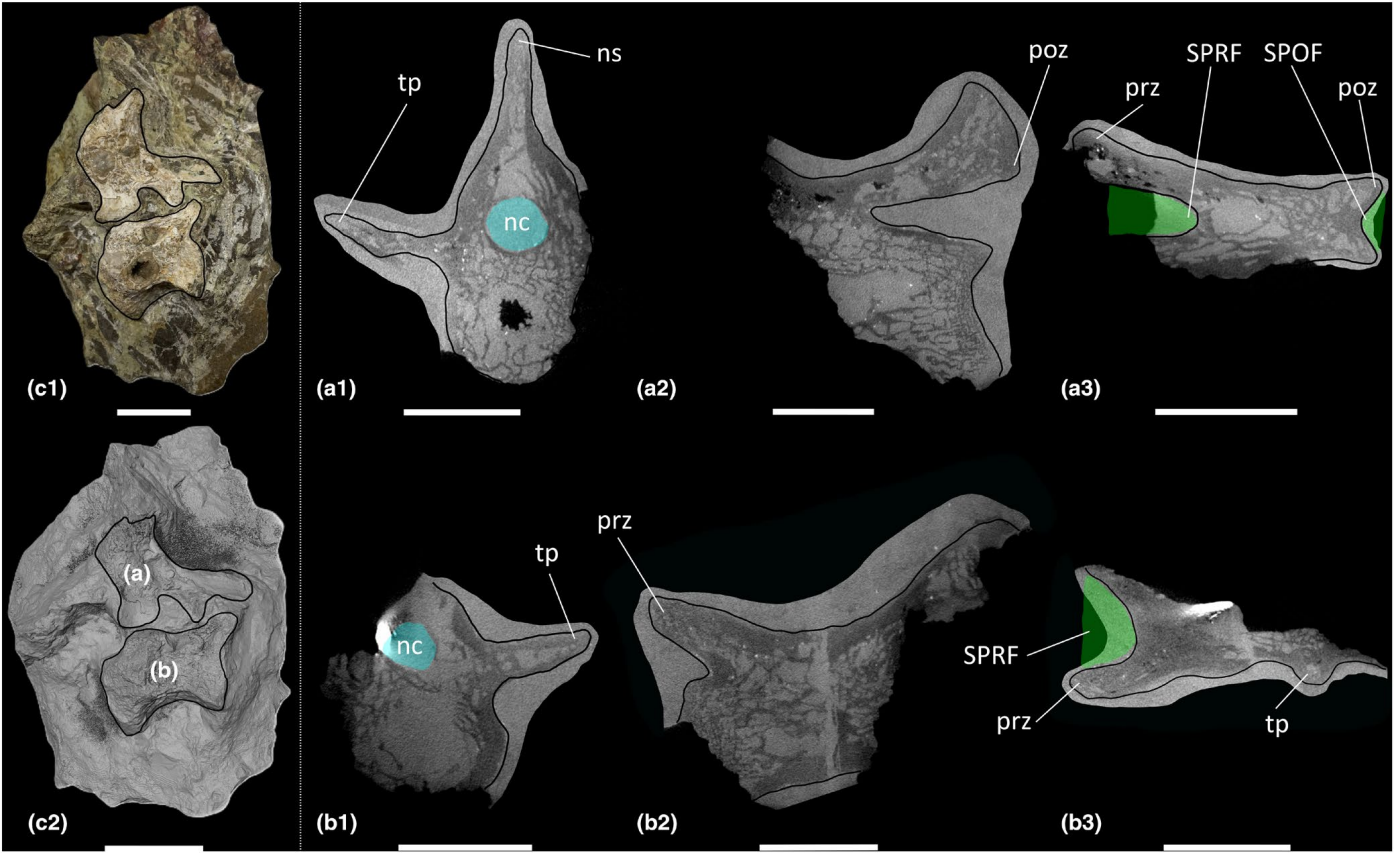
CT scan data of *Thecodontosaurus antiquus* caudal vertebrae in Cb 4164 block. (a) element A, (b) element B; (a1–b1) transverse cross‐section, (a2–b2) parasagittal cross‐section, (a3–b3) horizontal cross‐section; (c1) photograph of block, (c2) surface scan of block. nc, neural canal; ns, neural spine; poz, postzygapophysis; prz, prezygapophysis; SPOF, spinopostzygapophyseal fossa; SPRF, spinoprezygapophyseal fossa; tp, transverse process. Key: Blue highlight, neural canal; green highlight, fossa. Scale bars = 2 cm (a, b), 5 cm (c).

**FIGURE 4:**
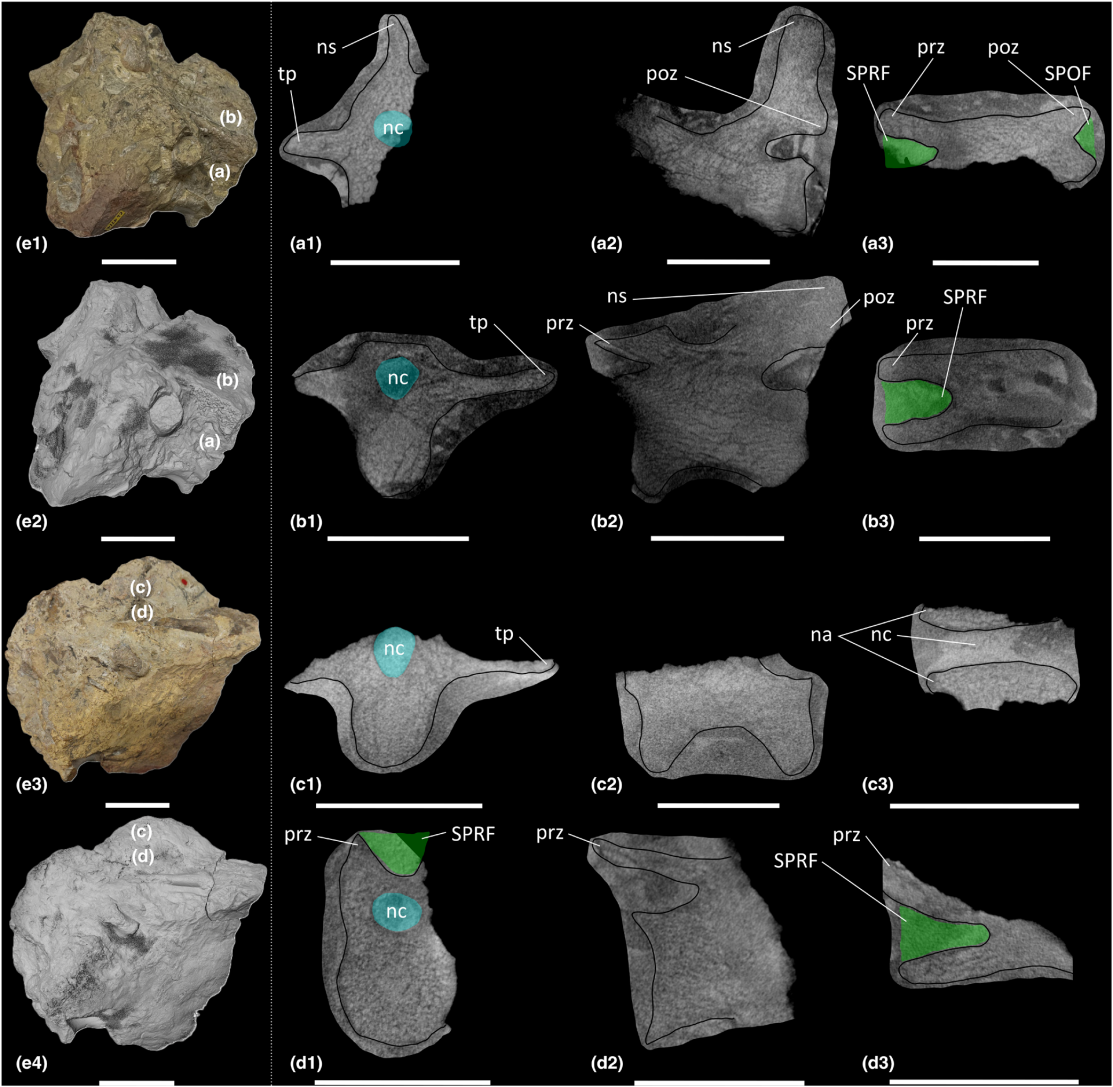
CT scan data of *Thecodontosaurus antiquus* caudal vertebrae in Cb 4714 block. (a) element A, (b) element B, (c) element C, (d) element D; (a1–d1) transverse cross‐section, (a2–d2) parasagittal cross‐section, (a3–d3) horizontal cross‐section; (e1–e2) side 1 of block, (e3–e4) side 2 of block; (e1, e3) photograph of block, (e2, e4) surface scan of block. na, neural arch; nc, neural canal; ns, neural spine; poz, postzygapophysis; prz, prezygapophysis; SPOF, spinopostzygapophyseal fossa; SPRF, spinoprezygapophyseal fossa; tp, transverse process. Key: Blue highlight, neural canal; green highlight, fossa. Scale bars = 2 cm (a–d), 5 cm (e).

The two CT scanned blocks (BRSMG Cb 4164, Figure 3; and BRSMG Cb 4714, Figure 4) collectively preserve six anterior–middle caudal vertebrae, all of which are incomplete, missing portions of their centra and neural arches. The approximate positions of the caudal vertebrae are based on the possession of transverse processes and a tall, dorsally projecting neural spine on the posterior half of each vertebra. However, as in the dorsal vertebrae, more accurate positions cannot be determined owing to their incompleteness. The anterior–middle caudal vertebrae possess a SPRF and SPOF, and their internal texture is spongious in the centra and neural arches. However, the centra of the two caudal vertebrae preserved in BRSMG Cb 4164 (elements ‘A’ and ‘B’) each possess a cavity located medially (Figure 3a1,b1). In element ‘A’ of BRSMG Cb 4164 (Figure 3a1,a2), this cavity extends along at least the posterior half of the centrum, although it does not extend as far as the posterior cotyle. Its anterior extent within the centrum cannot be determined owing to poor preservation. The centrum cavity in element B of BRSMG Cb 4164 (Figure 3b1,b2) might be a taphonomic artefact as it does not appear to extend anteroposteriorly. The neural arch of element ‘A’ of BRSMG Cb 4164 possesses some less densely packed trabeculae than in the centrum, just posterior to the SPRF, but this region does not appear to communicate externally (Figure 3a3).

### 3.2 Pantydraco caducus

#### 3.2.1 Cervical vertebrae

**FIGURE 5:**
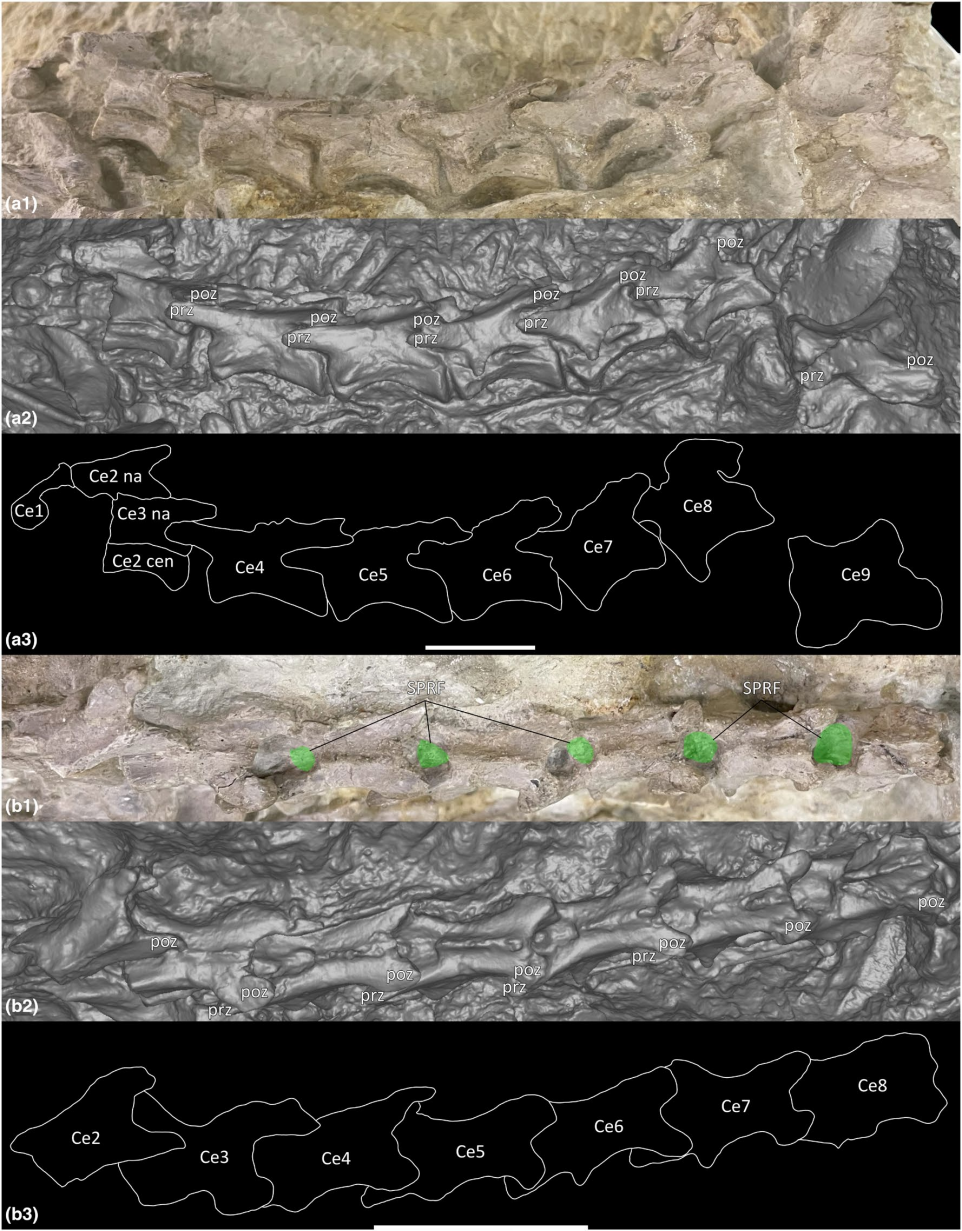
Cervical vertebral sequence of *Pantydraco caducus*. (a) Lateral view (a1) photograph, (a2) surface scan, (a3) outlined; (b) dorsal view (b1) photograph, (b2) surface scan, (b3) outlined. Ce, cervical vertebra; cen, centrum; na, neural arch; poz, postzygapophysis; prz, prezygapophysis; SPRF, spinoprezygapophyseal fossa. Key: Green highlight, fossa. Scale bars = 2 cm.

The holotype specimen of *Pantydraco* (NHMUK PV RUP24) preserves a complete cervical vertebral column (Ce1–Ce10; Yates, 2003a), but Ce10 could not be located in the NHMUK collection. Since the most recent description of *Pantydraco* (Galton & Kermack, 2010), the left prezygapophysis of Ce3 and the right diapophysis of Ce9 have been damaged (Figure 5a1,a2; cf. Galton & Kermack, 2010: fig. 13).

**FIGURE 6:**
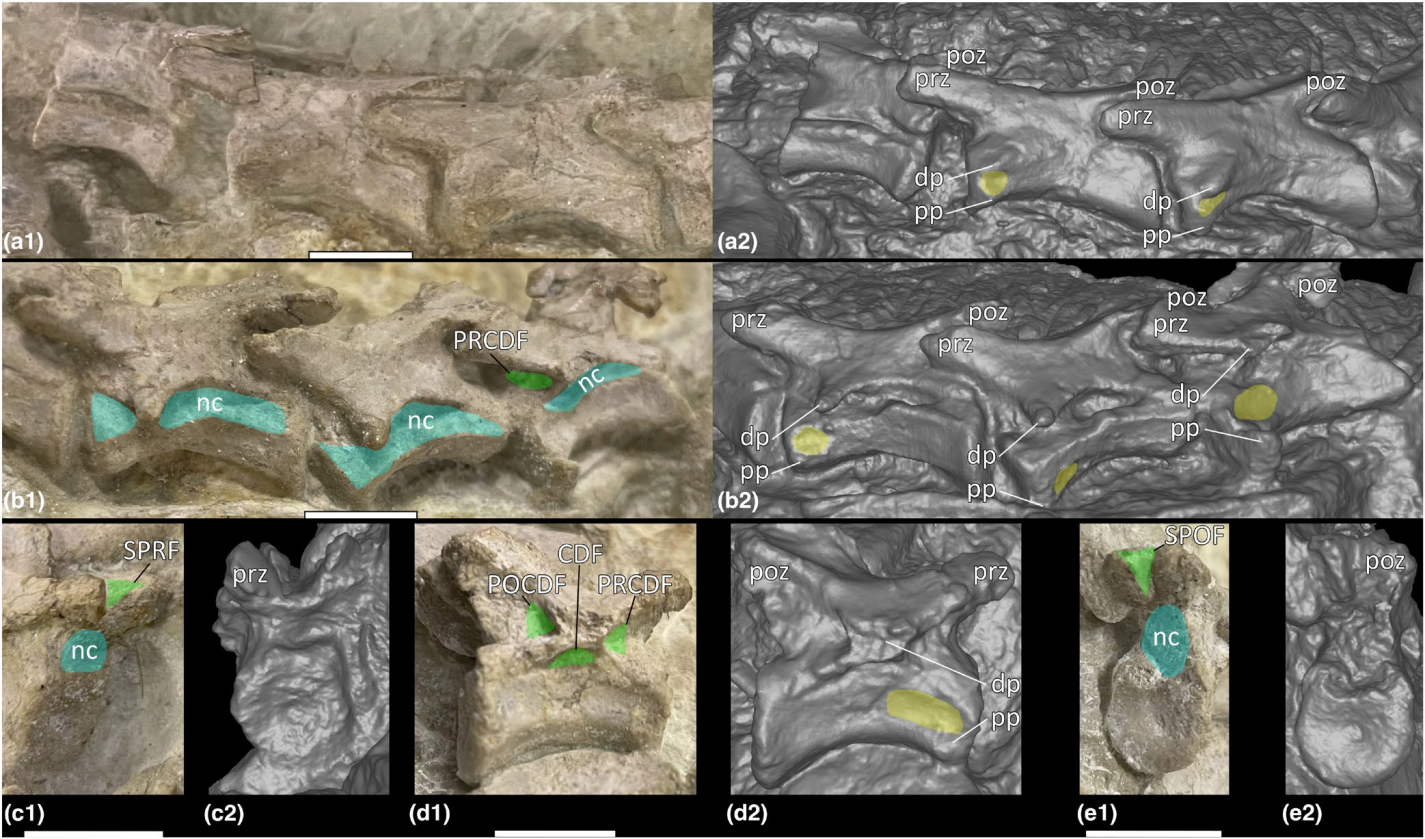
External features of cervical vertebral sequence of *Pantydraco caducus*. (a) lateral view of cervical centrum 2 and cervical neural arch 3–cervical vertebra 5 (a1) photograph, (a2) surface scan; (b) lateral view of cervical vertebra 6–cervical vertebra 8 (b1) photograph, (b2) surface scan; (c–e) cervical vertebra 9 (c1) anterolateral view photograph, (c2) anterior view surface scan, (d1) lateral view photograph, (d2) lateral view surface scan, (e1) posterior view photograph, (e2) posterior view surface scan. CDF, centrodiapophyseal fossa; dp, diapophysis; nc, neural canal; POCDF, postzygapophyseal centrodiapophyseal fossa; poz, postzygapophysis; pp., parapophysis; PRCDF, prezygapophyseal centrodiapophyseal fossa; prz, prezygapophysis; SPOF, spinopostzygapophyseal fossa; SPRF, spinoprezygapophyseal fossa. Key: Blue highlight, neural canal; green highlight, fossa; yellow highlight, concavity between parapophysis and diapophysis. Scale bars = 1 cm.

**FIGURE 7:**
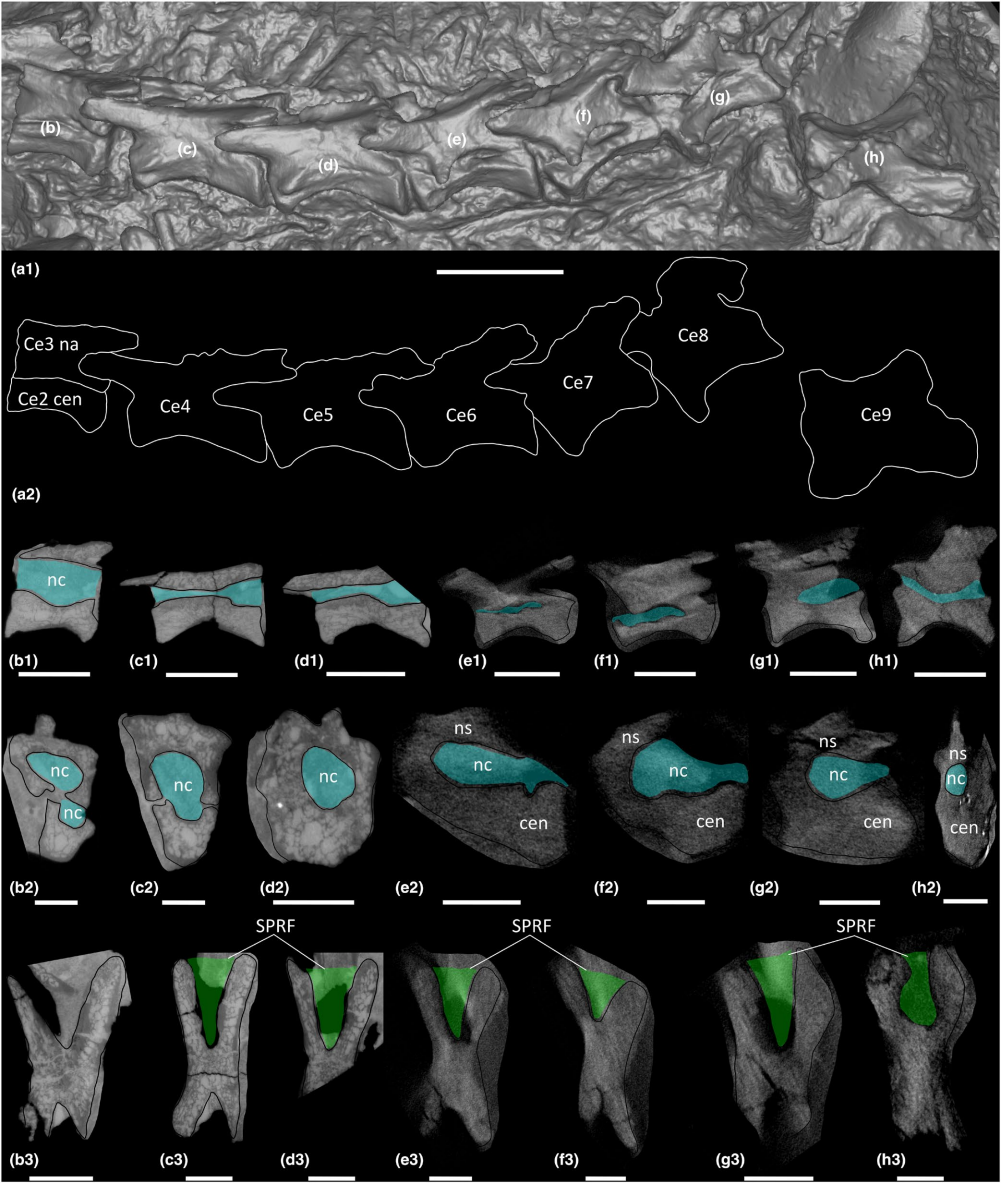
CT scan data of *Pantydraco caducus* cervical vertebrae. (a) lateral view of sequence (a1) surface scan, (a2) outlined; (b) cervical centrum 2 and cervical neural arch 3, (c) cervical vertebra 4, (d) cervical vertebra 5, (e) cervical vertebra 6, (f) cervical vertebra 7, (g) cervical vertebra 8, (h) cervical vertebra 9; (b1–h1) parasagittal cross‐section, (b2–h2) transverse cross‐section, (b3–h3) horizontal cross‐section. Ce, cervical vertebra; cen, centrum; nc, neural canal. Key: Blue highlight, neural canal; green highlight, fossa. Scale bars = 2 cm (a), 1 cm (b1–h1), 0.5 cm (b2–h3).

The lateral surfaces of the centra are shallowly concave anteroposteriorly but lack true lateral pneumatic fossae (LPF). On each element, the parapophysis is located at the ventrolateral edge of the anterior condyle. In Ce3–Ce5, the diapophysis is located just dorsal of the parapophysis. From Ce6 onwards, the diapophysis gradually migrates posterodorsally, becoming a prominent process. Between the parapophysis and diapophysis, there is a fossa located anteriorly on the centrum of each element (Figure 6a2,b2,d2). In Ce6–Ce7, this fossa is slightly ventrally offset in comparison to the other cervical vertebrae, owing to a left lateral rotation and ventral displacement of the centrum away from the neural arch. This rotation and displacement are evident from the exposed neurocentral suture, as previously described by Yates (2003a). However, the supposedly distinct sharp‐edged depressions or ‘pseudopleurocoels’ in Ce6–Ce8 described by Yates (2003a: fig. 12) (see also Wedel, 2007: fig. 10), located just ventral to the diapophyses, are actually part of the neural canal (Figure 6b1). The grey‐yellow colour of this area (i.e., matrix that has infilled the neural canal) in Ce6–Ce8 is the same as the matrix surrounding each bone in the holotype block. Conversely, the colour of each bone is a distinct pale pink‐grey colour. This observation is further upheld from CT scan data wherein the transverse cross‐section of the offset between the centra and neural arches of Ce6–Ce8 can be seen, along with the unenclosed neural canal (Figure 7e2,f2,g2).

Ce8–Ce10 each possess a PRCDF and CDF, and a POCDF and SPOF are present on Ce9–Ce10 (Figure 6b1,d1,e1; Galton & Kermack, 2010: fig. 14). Ce4–Ce10 each possess an SPRF, but the presence or absence of an SPRF in Ce3 cannot be assessed because the corresponding area is covered by the postzygapophysis of Ce2 (Figures 5b1 and 6c1; Galton & Kermack, 2010: fig. 14). CT scans reveal spongious internal texture in the centra and neural arches (Figure 7).

### 3.3 Ruehleia bedheimensis

#### 3.3.1 Cervical vertebrae

With the exception of the atlas, *Ruehleia* is known from a complete cervical vertebral series (Ce2–Ce10; MB.R.4718.20, MB.R.4718.50–57). It is possible that Ce10 is instead the first dorsal vertebra because the morphology of the neural arch is more similar to that of the dorsal vertebrae than the cervical vertebrae.

Each cervical centrum possesses anteroposteriorly concave lateral and ventral surfaces. However, the lateral surfaces lack a true LPF. Ce3–Ce6 lack neural arch fossae, and Ce7–Ce10 possess a shallow PRCDF and CDF ventral to the diapophysis, separated by a faint anterior centrodiapophyseal lamina (ACDL). Only Ce9–Ce10 possess a POCDF. Each cervical neural spine possesses a SPRF and SPOF, with both fossae being deepest in Ce10.

**FIGURE 8:**
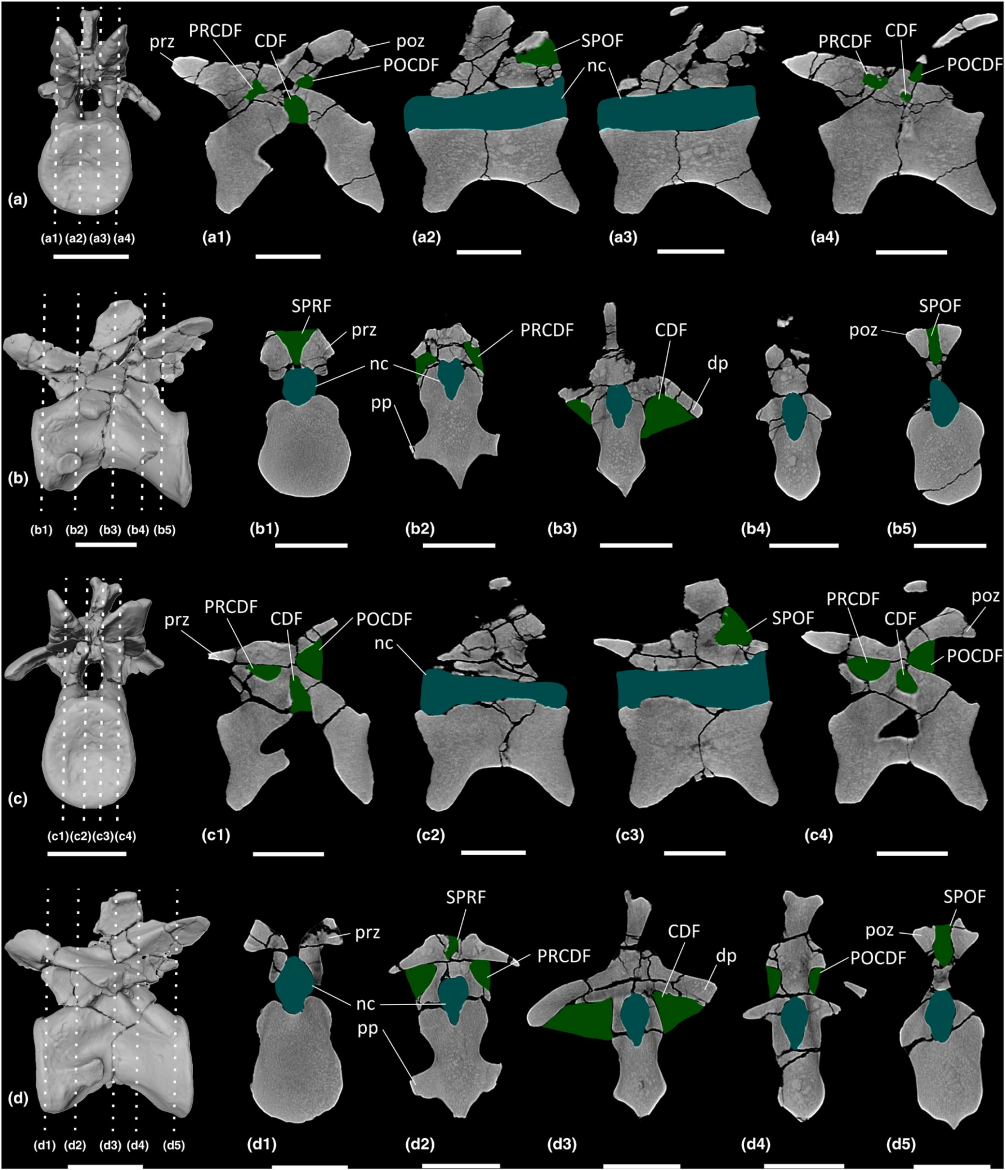
CT scan data of *Ruehleia bedheimensis* cervical vertebrae. (a, b) cervical vertebra 9 MB.R.4718.56, (c, d) cervical vertebra 10 MB.R.4718.57; (a, c) surface scan anterior view, (b, d) surface scan lateral view; (a1–a4, c1–c4) CT scan slices in parasagittal cross‐section, (b1–b5, d1–d5) CT scan slices in transverse cross‐section. CDF, centrodiapophyseal fossa; dp, diapophysis; nc, neural canal; POCDF, postzygapophyseal centrodiapophyseal fossa; poz, postzygapophysis; pp, parapophysis; PRCDF, prezygapophyseal centrodiapophyseal fossa; prz, prezygapophysis; SPOF, spinopostzygapophyseal fossa; SPRF, spinoprezygapophyseal fossa. Key: Blue highlight, neural canal; green highlight, fossa. Scale bars = 5 cm.

CT scans of Ce9 and Ce10 demonstrate that their centra possess spongious internal texture composed of dense trabeculae (Figure 8). The centra of Ce9–Ce10 possess centrally clustered cavities (Figure 8a2–a3,b4,c3,d3,d4). The neural arches of Ce9–Ce10 are characterised by less densely packed trabeculae than their respective centra. The SPOF communicates with small, clustered cavities that extend anteroposteriorly through the neural spines, but these do not extend dorsally to the neural spine apex (Figure 8a2,a3,b2–b4,c2,c3,d2–d5). These cavities are more numerous in Ce9 than in Ce10.

#### 3.3.2 Cervical ribs

**FIGURE 9:**
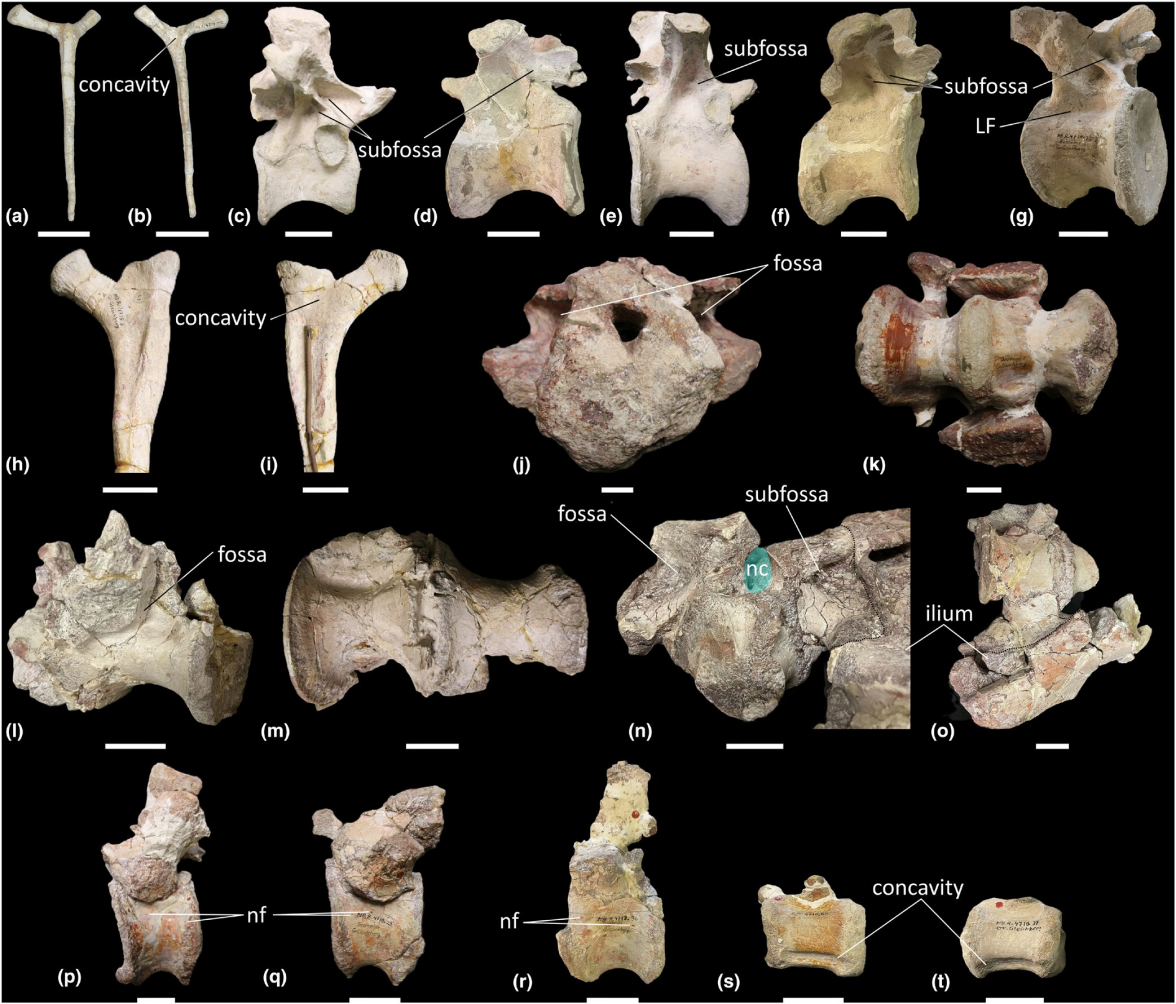
Photographs of axial elements of *Ruehleia bedheimensis*. (a, b) cervical rib MB.R.4718.112 (a) lateral view, (b) medial view; (c) right lateral view of dorsal vertebra 5; (d) left lateral view of dorsal vertebra 9; (e) right lateral view of dorsal vertebra 10; (f) left lateral view of dorsal vertebra 13; (g) left lateral view of dorsal vertebra 15; (h, i) dorsal rib MB.R.4718.3 (h) anterior view, (i) posterior view; (j, k) holotype sacrum MB.R.4718.27 (j) posterior view, (k) ventral view; (l, m) referred sacrum MB.R.4774 (l) lateral view, (m) ventral view; (n, o) referred sacrum MB.R.6751 (n) posterior view, (o) ventral view; (p–r) anterior–middle caudal vertebrae in left lateral view (p) MB.R.4718.28, (q) MB.R.4718.29, (r) MB.R.4718.36; (s, t) ventrolateral view of middle–posterior caudal vertebrae (s) MB.R.4718.65, (t) MB.R.4718.37. LF, lateral fossa; nc, neural canal; nf, nutrient foramen. Key: Blue highlight, neural canal. Scale bars = 5 cm.

One complete cervical rib (MB.R.4718.112) and several cervical rib fragments are preserved (Figure 9a,b). The former possesses a shallow concavity on the medial surface between the capitulum and tuberculum (Figure 9b). The cervical rib fragments reveal a solid internal bone texture along the entire length of the shaft.

#### 3.3.3 Dorsal vertebrae

A complete dorsal vertebral series (D1–D15; MB.R.4718.41‐49, MB.R.4718.67‐72) is preserved. As in the cervical vertebrae, the ventral surfaces are anteroposteriorly concave. The lateral surfaces of the centra are more deeply concave than those of the cervical vertebrae. Just ventral to the base of the neural canal, D15 possesses a lateral fossa that is not a true LPF (Figure 9g). Within this lateral fossa, there is a small, prominent nutrient foramen, and several smaller nutrient foramina appear on both sides of the centrum. However, the centrum of D15 has been restored with plaster and the right side is missing some external bone. Therefore, the exact number and dimensions of the nutrient foramina cannot be determined with certainty. Nutrient foramina also pierce the lateral surfaces of several other dorsal centra, and D5 possesses a nutrient foramen on the right lateral surface, close to the dorsal tip of the neural spine.

D1–D7 possess the same fossae as the posterior cervical vertebrae Ce9–Ce10, but they are deeper in the dorsal vertebrae. The PRCDF becomes progressively dorsoventrally shorter along the dorsal vertebral sequence and is absent from D8 onwards as a result of the dorsal migration of the parapophysis. D14–D15 each possess a parapophyseal centroprezygapophyseal fossa (PACPRF) on the anterior surface of the neural arch, ventrolateral to the prezygapophyses, level with the roof of the neural canal. This fossa is deeper in D15. Several fossae are subdivided by an accessory lamina that occurs on only one side of the element: the right PRCDF of D5; the right CDF of D5 and D10; the left CDF of D13; and the left POCDF of D9, D13 and D15 (Figure 9c–g).

**FIGURE 10:**
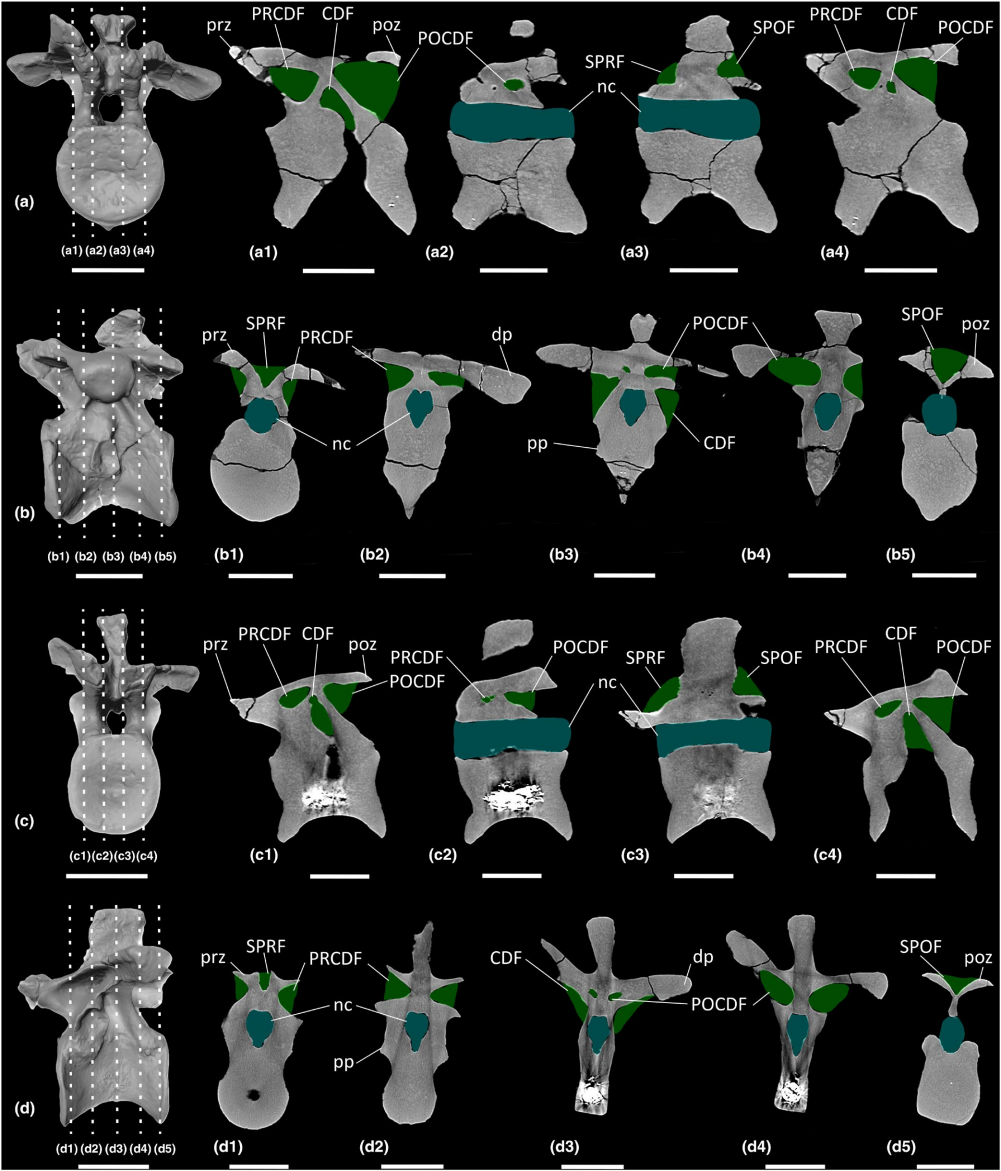
CT scan data of *Ruehleia bedheimensis* dorsal vertebrae. (a, b) dorsal vertebra 1 MB.R.4718.41, (c, d) dorsal vertebra 5 MB.R.4718.45; (a, c) surface scan anterior view, (b, d) surface scan lateral view; (a1–a4, c1–c4) CT scan slices in parasagittal cross‐section, (b1–b5, d1–d5) CT scan slices in transverse cross‐section. CDF, centrodiapophyseal fossa; dp, diapophysis; nc, neural canal; POCDF, postzygapophyseal centrodiapophyseal fossa; poz, postzygapophysis; pp, parapophysis; PRCDF, prezygapophyseal centrodiapophyseal fossa; prz, prezygapophysis; SPOF, spinopostzygapophyseal fossa; SPRF, spinoprezygapophyseal fossa. Key: Blue highlight, neural canal; green highlight, fossa. Scale bars = 5 cm.

The dorsal centra and neural arches possess apneumatic spongious internal texture composed of dense trabecular bone. CT scans of D1 and D5 reveal that the centrum of D1 possesses some larger cavities centrally that do not communicate externally (Figure 10a2,b4). Unfortunately, the centrum of D5 appears to possess a metal rod or other conservation material that reduces the contrast resolution of the CT scan in this region due to beam hardening (Figure 10c1–c3,d3,d4). The neural arches of D1 and D5 possess some small, clustered cavities that do not communicate with any external fossae. Additionally, the subdivided right PRCDF and CDF of D5 do not communicate with any internal cavities.

#### 3.3.4 Dorsal ribs

Many complete dorsal ribs and dorsal rib fragments are preserved (MB.R.4718.1‐19, MB.R.4718.21‐25, Figure 9h,i). Similar to the complete cervical rib, there is a shallow concavity on the posterior surface between the capitulum and tuberculum (Figure 9i). Worn surfaces and broken surfaces reveal a solid internal bone texture along the shaft.

#### 3.3.5 Sacrum

Three partial sacra have been assigned to *Ruehleia*: the holotype sacrum (MB.R.4718.27, Figure 9j,k), which consists of two fused centra with neural arch bases; two incomplete fused sacral centra with neural arch bases (MB.R.4774, Figure 9l,m); and an S1 centrum with neural arch base that is fused to a partial right ilium (MB.R.6751, Figure 9n,o). Unless otherwise stated, the following description is based on all three sacra.

Broken and eroded surfaces reveal a spongious internal texture in the centra and neural arch bases. The lateral surfaces of the centra are anteroposteriorly concave, whereas the ventral surfaces are transversely convex and shallowly concave anteroposteriorly. The posterior surface of each transverse process possesses a fossa that is level with the neural canal (Figure 9j,l,n). On the right side of S1 of MB.R.6751, this fossa is subdivided by a dorsomedially–ventrolaterally oriented lamina (Figure 9n). No further pneumatic characteristics of the neural arches can be assessed because of incompleteness.

#### 3.3.6 Caudal vertebrae

Nineteen caudal vertebrae that are not a sequential series are preserved (MB.R.4718.28‐38, MB.R.4718.60‐66, MB.R.4718.113). Anterior, middle, and posterior positions in the tail sequence are designated based on size and the extent of the transverse processes, but it is not possible to more accurately determine caudal vertebral placements.

The lateral and ventral surfaces of the centra are shallowly concave anteroposteriorly, and the lateral surfaces lack a LPF. Three anterior–middle caudal centra (MB.R.4718.28‐30) possess small nutrient foramina on the dorsal one‐third of their left lateral surface (Figure 9p–r). Two middle–posterior caudal centra (MB.R.4718.37, MB.R.4718.65) possess a shallow, anteroposteriorly elongate ventral concavity that is bounded by ventrolateral ridges (Figure 9s,t). Broken and eroded surfaces reveal spongious internal texture in both the centra and neural arches.

### 3.4 Plateosaurus longiceps

#### 3.4.1 Cervical vertebrae

**FIGURE 11:**
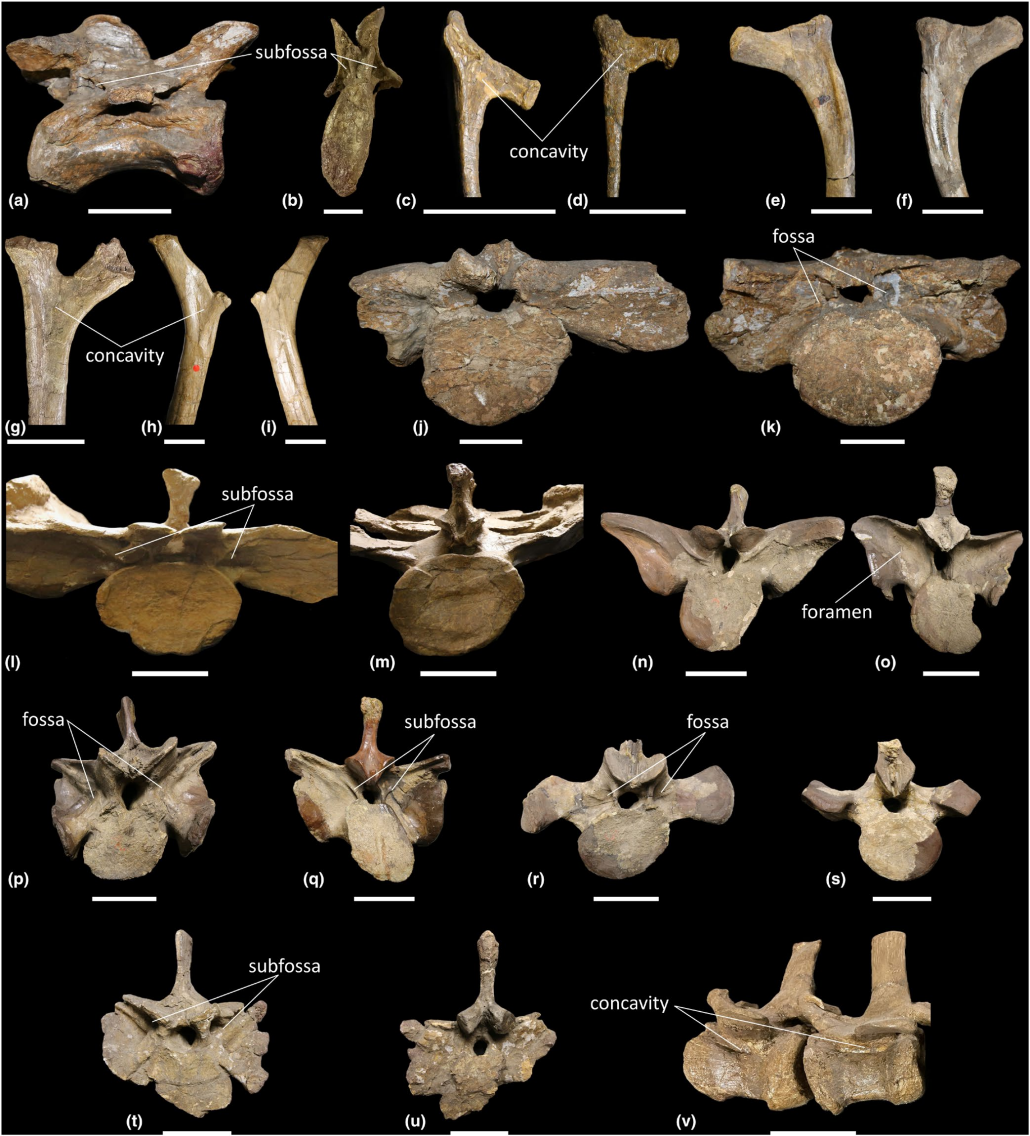
Photographs of axial elements of *Plateosaurus longiceps*. (a) right lateral view of cervical vertebra 9 MB.R.4404.23 ‘Skeleton 25’; (b) anterior view of cervical vertebra 10 MB.R.4430.21 ‘Skeleton C’; (c, d) medial view of ‘Skeleton C’ cervical ribs (c) MB.R.4430.181, (d) MB.R.4430.104; (e, f) dorsal rib MB.R.4405.10 ‘Skeleton 1’ (e) anterior view, (f) posterior view; (g) posterior view of dorsal rib MB.R.4430.119 ‘Skeleton C’; (h, i) dorsal rib MB.R.4430.110 ‘Skeleton C’ (h) posterior view, (i) anterior view; (j, k) sacrum MB.R.4398.25 ‘Skeleton 42’ (j) anterior view of sacral vertebra 1, (k) posterior view of sacral vertebra 2; (l, m) sacrum MB.R.4430.37 ‘Skeleton C’ (l) anterior view of sacral vertebra 1, (m) posterior view of sacral vertebra 3; (n–s) sacrum MB.R.4405 ‘Skeleton 1’ (n, o) sacral vertebra 1 MB.R.4405.12, (p, q) sacral vertebra 2 MB.R.4405.13, (r, s) sacral vertebra 3 MB.R.4405.85, (n, p, r) anterior view, (o, q, s) posterior view; (t, u) sacral vertebra 2 MB.R.4392 (t) anterior view, (u) posterior view; (v) left lateral view of caudal vertebrae 13–14 MB.R.4430.50–51 ‘Skeleton C’. Scale bars = 5 cm (a–i), 10 cm (j–v).

The following description is based on the complete cervical vertebral series of ‘Skeleton C’ (MB.R.4430.13‐21) and ‘Skeleton 25’ (MB.R.4404.16‐24), with each consisting of 10 vertebrae. The lateral surfaces of the cervical vertebrae are shallowly concave anteroposteriorly, but lack a LPF. Ce6–Ce10 possess a CDF that becomes deeper along the series. Ce9–Ce10 possess a PRCDF and POCDF, each of which is deeper and more pronounced in the latter vertebra. In Ce9 of ‘Skeleton 25’ (MB.R.4404.23), the right POCDF is subdivided (Figure 11a), and the CDF is deeper than that in Ce9 of ‘Skeleton C’ (MB.R.4430.20), owing to a more pronounced posterior centrodiapophyseal lamina (PCDL) in the former. The PRCDF of Ce10 of ‘Skeleton C’ (MB.R.4430.21) is subdivided on both the left and right sides (Figure 11b). Neural spines of all cervical vertebrae possess an SPRF and SPOF.

**FIGURE 12:**
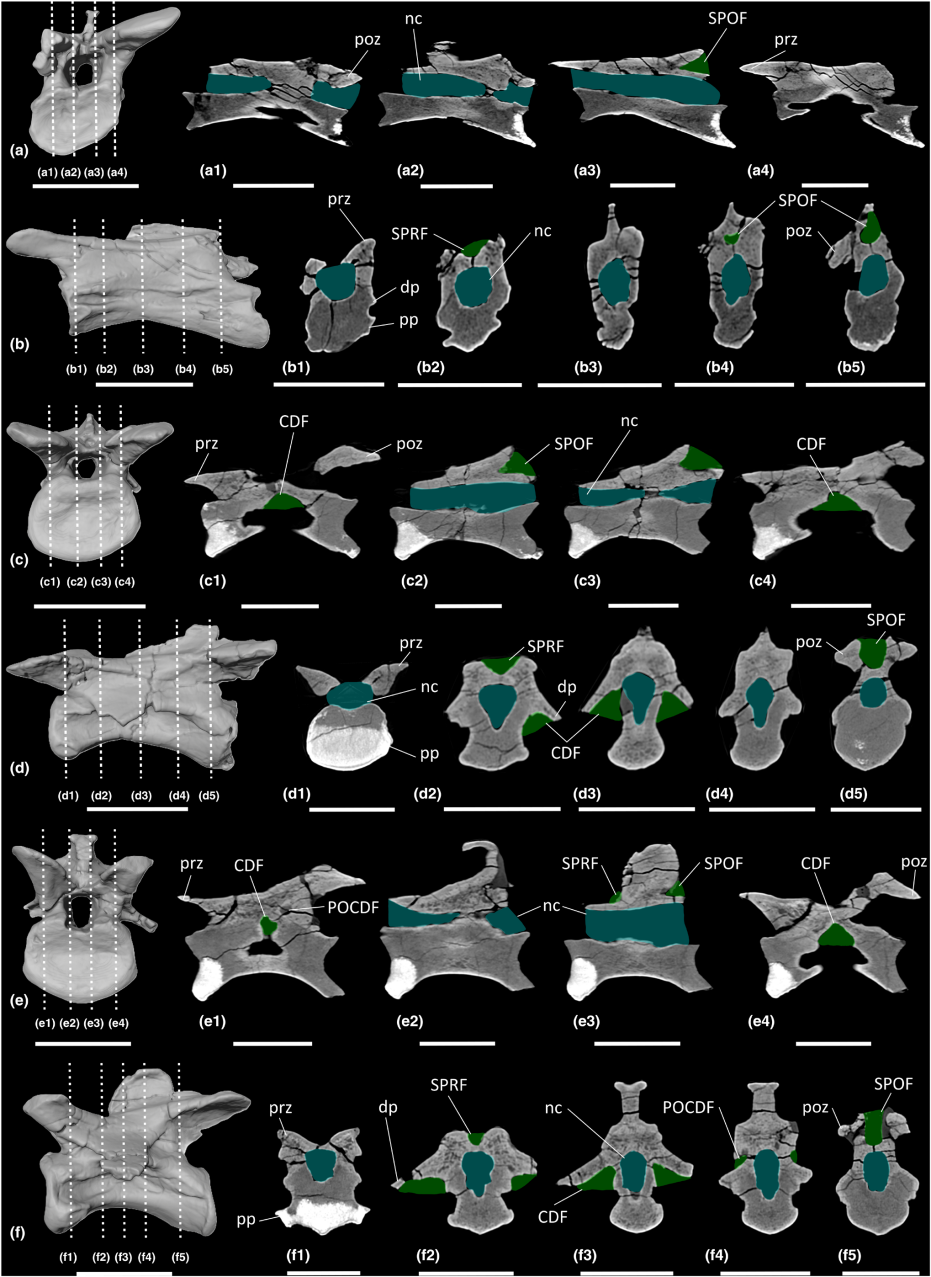
CT scan data of *Plateosaurus longiceps* MB.R.4404 ‘Skeleton 25’ cervical vertebrae. (a, b) cervical vertebra 4 MB.R.4404.18, (c, d) cervical vertebra 7 MB.R.4404.21, (e, f) cervical vertebra 9 MB.R.4404.23; (a, c, e) surface scan anterior view, (b, d, f) surface scan lateral view; (a1–a4, c1–c4, e1–e4) CT scan slices in parasagittal cross‐section, (b1–b5, d1–d5, f1–f5) CT scan slices in transverse cross‐section. CDF, centrodiapophyseal fossa; dp, diapophysis; nc, neural canal; POCDF, postzygapophyseal centrodiapophyseal fossa; poz, postzygapophysis; pp., parapophysis; prz, prezygapophysis; SPOF, spinopostzygapophyseal fossa; SPRF, spinoprezygapophyseal fossa. Key: Blue highlight, neural canal; green highlight, fossa. Scale bars = 5 cm.

**FIGURE 13:**
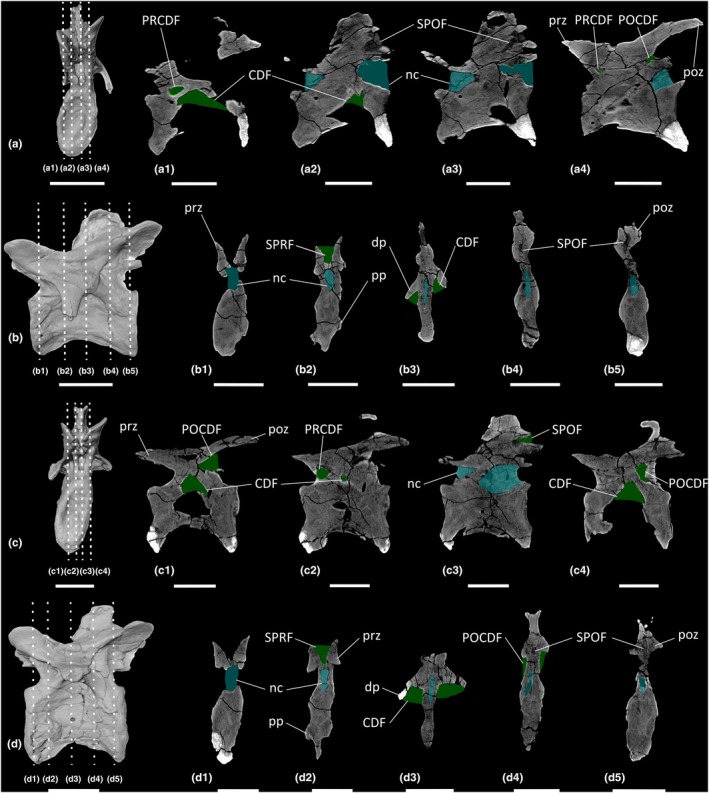
CT scan data of *Plateosaurus longiceps* MB.R.4430 ‘Skeleton C’ cervical vertebrae. (a, b) Cervical vertebra 9 MB.R.4430.20, (c, d) cervical vertebra 10 MB.R.4430.21; (a, c) surface scan anterior view, (b, d) surface scan lateral view; (a1–a4, c1–c4) CT scan slices in parasagittal cross‐section, (b1–b5, d1–d5) CT scan slices in transverse cross‐section. CDF, centrodiapophyseal fossa; dp, diapophysis; nc, neural canal; POCDF, postzygapophyseal centrodiapophyseal fossa; poz, postzygapophysis; pp, parapophysis; PRCDF, prezygapophyseal centrodiapophyseal fossa; prz, prezygapophysis; SPOF, spinopostzygapophyseal fossa; SPRF, spinoprezygapophyseal fossa. Key: Blue highlight, neural canal; green highlight, fossa. Scale bars = 5 cm.

The cervical centra possess apneumatic spongious internal texture. CT scans of anterior (Ce4), middle (Ce7) and posterior (Ce9–Ce10) cervical vertebrae reveal that the centra of the middle–posterior elements possess some clustered cavities, located ventromedially, that do not communicate with external fossae (Figures 12 and 13). The neural arches of all cervical vertebral elements possess less densely packed trabeculae than in the centra, and there are clustered cavities that extend anteroposteriorly, but not dorsally (Figures 12 and 13). These cavities are more clearly defined in ‘Skeleton 25’ (Figure 12) than in ‘Skeleton C’ (Figure 13), potentially owing to the transverse compression of the latter. Ce4 does not possess as many cavities as Ce7 and Ce9, nor are they as large. In Ce4, the clustered cavities do not communicate with any external fossae. However, in all middle–posterior elements of both cervical series, the CDF and SPOF do communicate with the internal cavities. The subdivided right POCDF of Ce9 of ‘Skeleton 25’ (MB.R.4404.23) also communicates with internal cavities (Figure 12e1,f4). Conversely, the subdivided left and right PRCDF of Ce10 of ‘Skeleton C’ (MB.R.4430.21) do not communicate with any internal cavities (Figure 13c2,c4).

#### 3.4.2 Cervical ribs

‘Skeleton C’ preserves several cervical ribs with varying degrees of completeness (MB.R.4430.96, MB.R.4430.98–104, MB.R.4430.181, MB.R.4430.205). Those that preserve proximal ends possess a shallow concavity on the medial surface between the tuberculum and capitulum (Figure 11c,d). The internal texture of the cervical ribs cannot be determined as they have been covered with clay and painted.

#### 3.4.3 Dorsal vertebrae

The dorsal vertebral series of ‘Skeleton C’ comprises D1–D15 (MB.R.4430.22–36), which likely represents the complete dorsal vertebral count (Nau et al., 2020; Regalado Fernández et al., 2023; Schaeffer, 2024), whereas the series of ‘Skeleton 25’ preserves only D1–D12 (MB.R.4404.25–36). The dorsal vertebrae possess the same fossae as in the posterior cervical vertebrae, but they are deeper in the dorsal vertebral series. As in the posterior cervical vertebrae, the dorsal centra possess shallowly anteroposteriorly concave lateral surfaces that lack a true LPF. As a result of the dorsally migrating parapophysis, the PRCDF becomes smaller and shallower along the sequence and is entirely absent in D8–D15.

**FIGURE 14:**
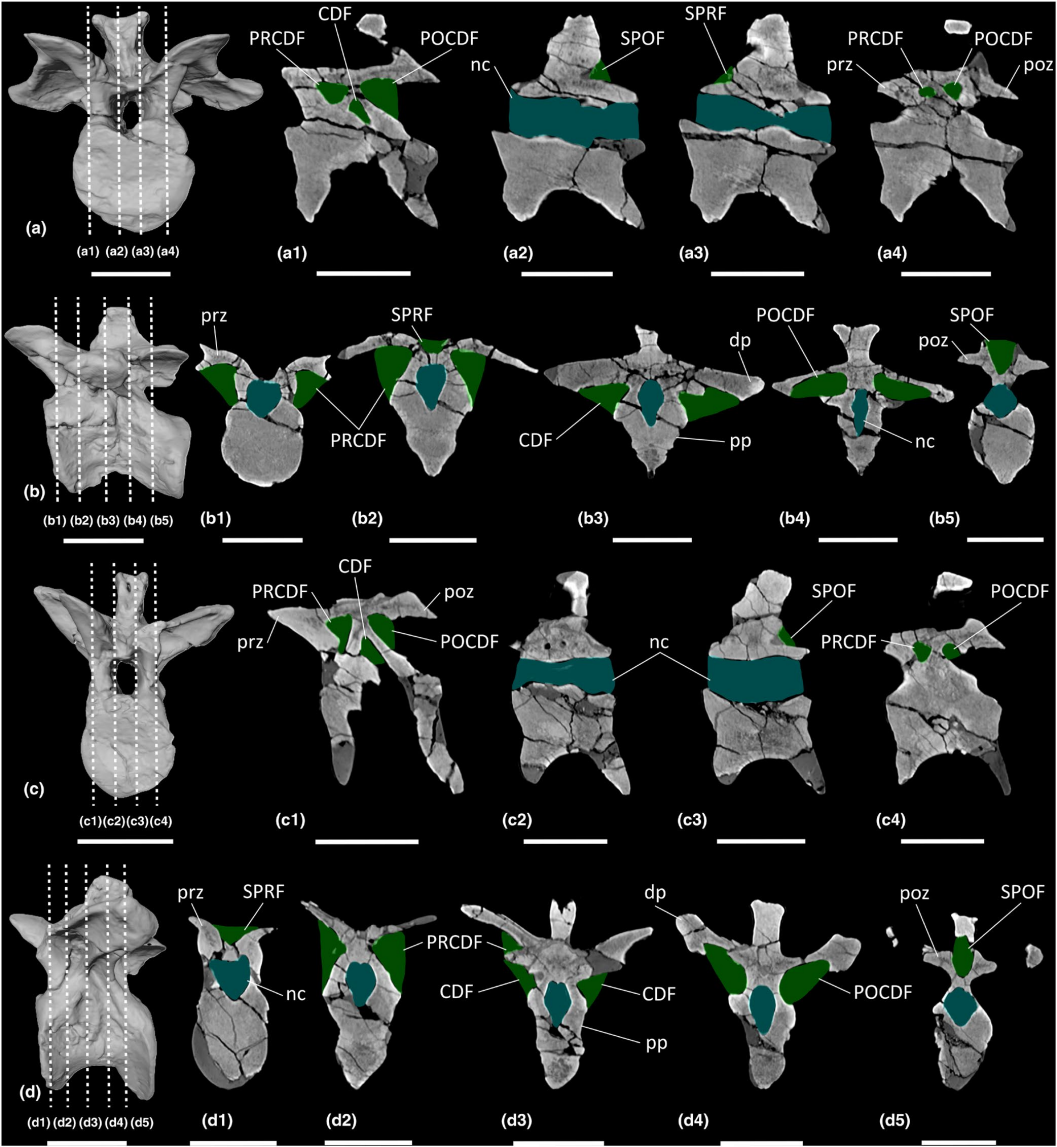
CT scan data of *Plateosaurus longiceps* MB.R.4404 ‘Skeleton 25’ anterior dorsal vertebrae. (a, b) dorsal vertebra 1 MB.R.4404.25, (c, d) dorsal vertebra 3 MB.R.4404.27; (a, c) surface scan anterior view, (b, d) surface scan lateral view; (a1–a4, c1–c4) CT scan slices in parasagittal cross‐section, (b1–b5, d1–d5) CT scan slices in transverse cross‐section. CDF, centrodiapophyseal fossa; dp, diapophysis; nc, neural canal; POCDF, postzygapophyseal centrodiapophyseal fossa; poz, postzygapophysis; pp, parapophysis; PRCDF, prezygapophyseal centrodiapophyseal fossa; prz, prezygapophysis; SPOF, spinopostzygapophyseal fossa; SPRF, spinoprezygapophyseal fossa. Key: Blue highlight, neural canal; green highlight, fossa. Scale bars = 5 cm.

**FIGURE 15:**
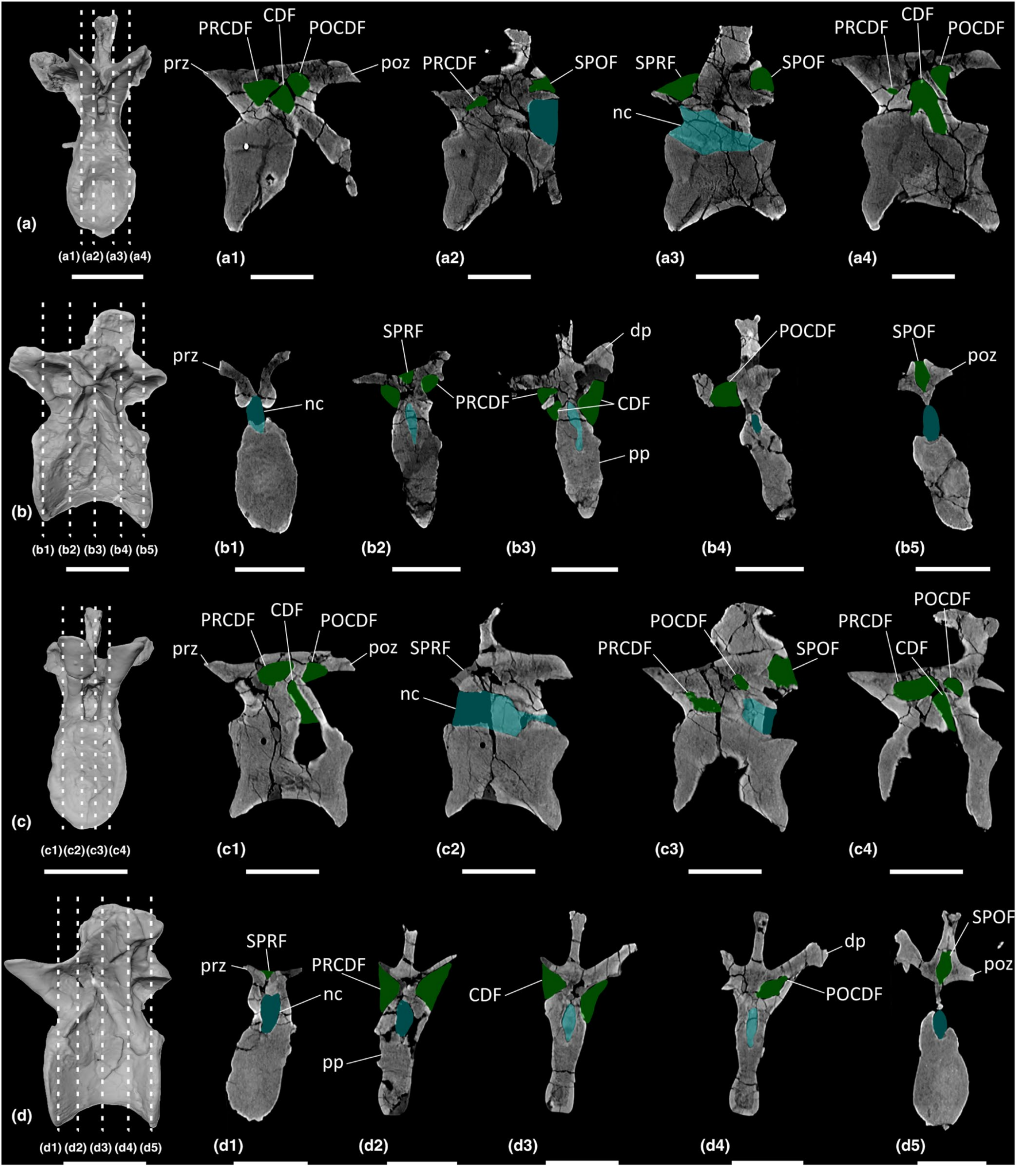
CT scan data of *Plateosaurus longiceps* MB.R.4430 ‘Skeleton C’ anterior dorsal vertebrae. (a, b) dorsal vertebra 2 MB.R.4430.23, (c, d) dorsal vertebra 4 MB.R.4430.25; (a, c) surface scan anterior view, (b, d) surface scan lateral view; (a1–a4, c1–c4) CT scan slices in parasagittal cross‐section, (b1–b5, d1–d5) CT scan slices in transverse cross‐section. CDF, centrodiapophyseal fossa; dp, diapophysis; nc, neural canal; POCDF, postzygapophyseal centrodiapophyseal fossa; poz, postzygapophysis; pp, parapophysis; PRCDF, prezygapophyseal centrodiapophyseal fossa; prz, prezygapophysis; SPOF, spinopostzygapophyseal fossa; SPRF, spinoprezygapophyseal fossa. Key: Blue highlight, neural canal; green highlight, fossa. Scale bars = 5 cm.

**FIGURE 16:**
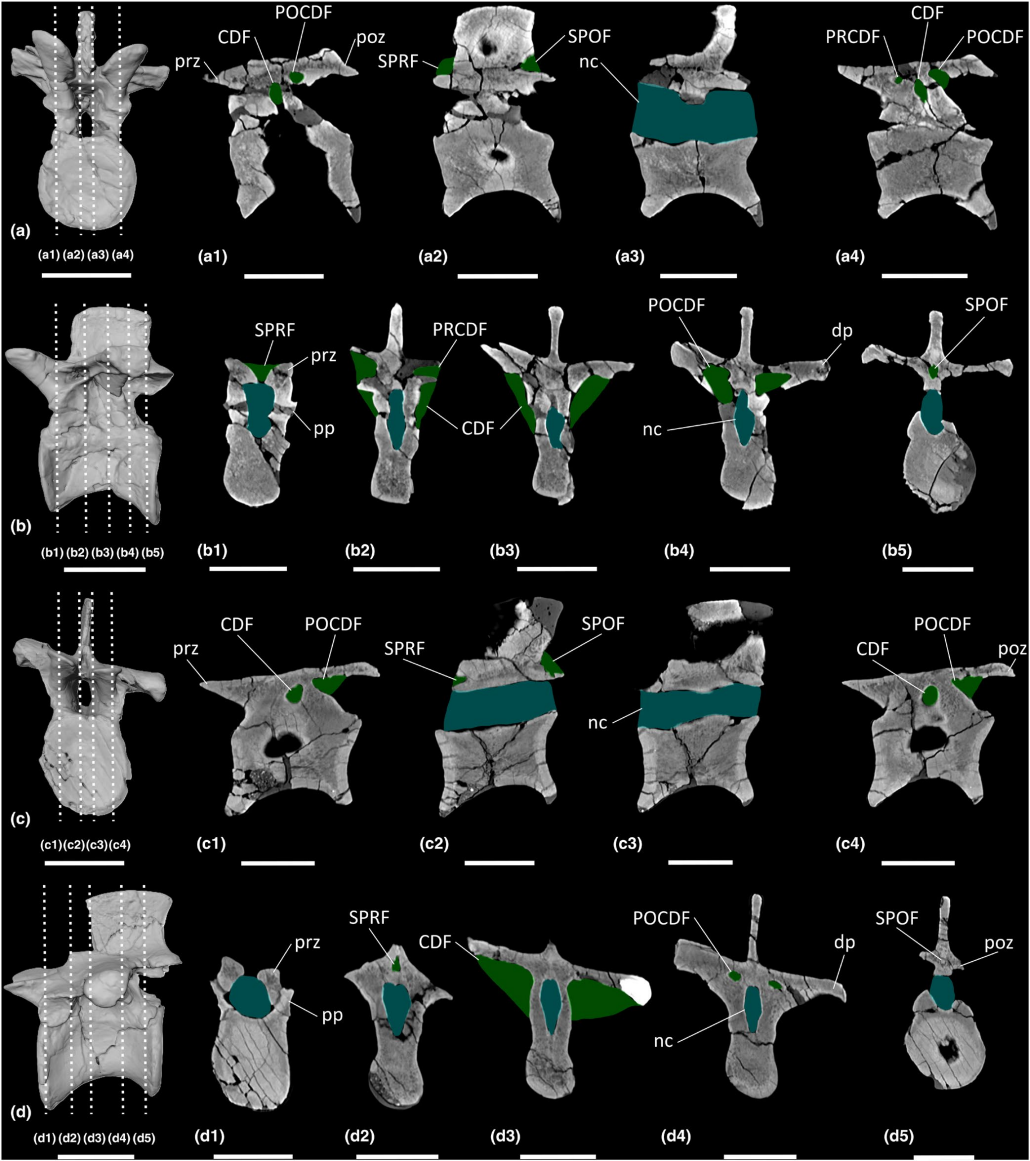
CT scan data of *Plateosaurus longiceps* MB.R.4404 ‘Skeleton 25’ middle dorsal vertebrae. (a, b) dorsal vertebra 6 MB.R.4404.30, (c, d) dorsal vertebra 10 MB.R.4404.34; (a, c) surface scan anterior view, (b, d) surface scan lateral view; (a1–a4, c1–c4) CT scan slices in parasagittal cross‐section, (b1–b5, d1–d5) CT scan slices in transverse cross‐section. CDF, centrodiapophyseal fossa; dp, diapophysis; nc, neural canal; POCDF, postzygapophyseal centrodiapophyseal fossa; poz, postzygapophysis; pp, parapophysis; PRCDF, prezygapophyseal centrodiapophyseal fossa; prz, prezygapophysis; SPOF, spinopostzygapophyseal fossa; SPRF, spinoprezygapophyseal fossa. Key: Blue highlight, neural canal; green highlight, fossa. Scale bars = 5 cm.

**FIGURE 17:**
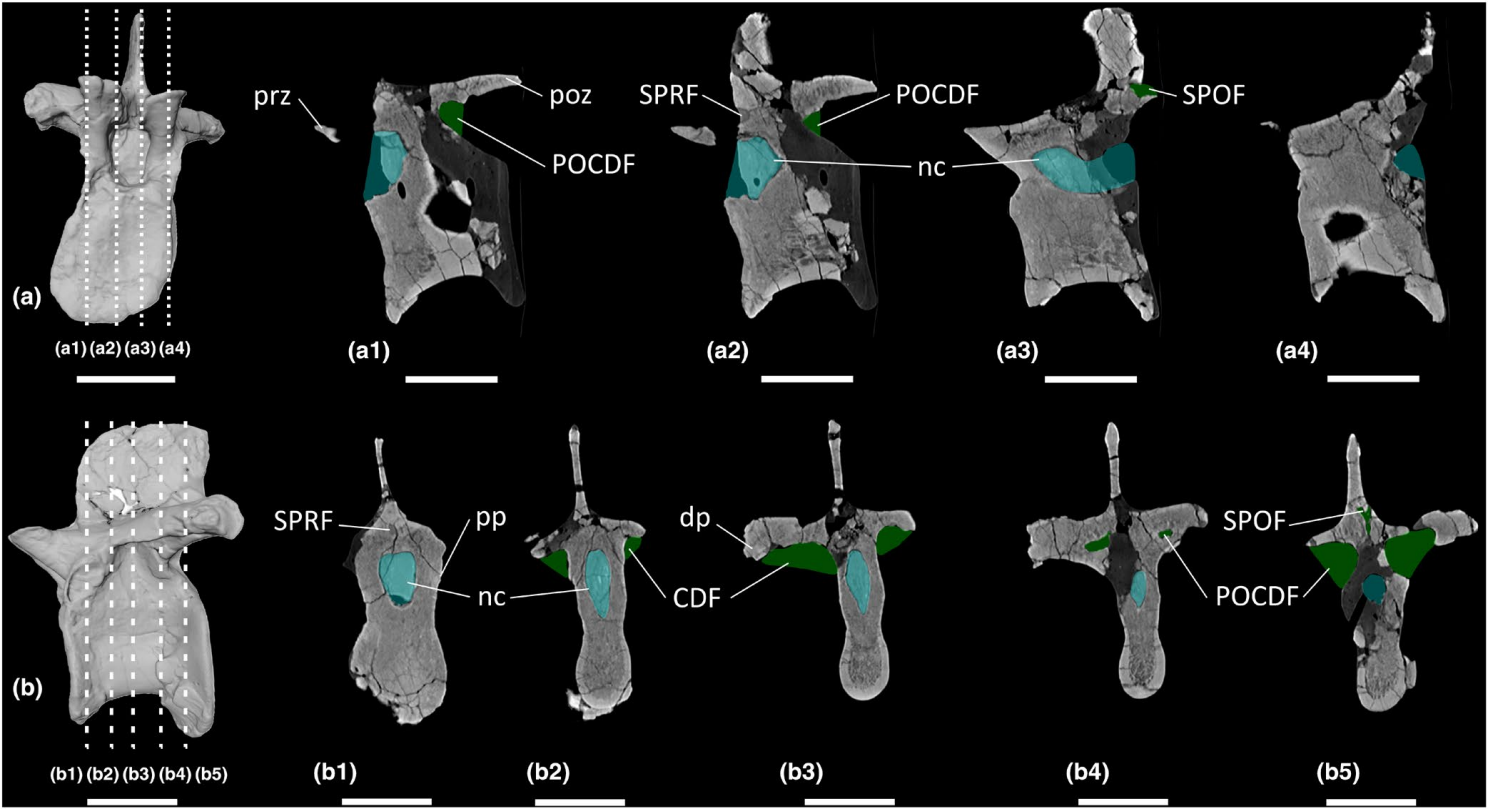
CT scan data of *Plateosaurus longiceps* MB.R.4430 ‘Skeleton C’ middle dorsal vertebra. (a, b) dorsal vertebra 9 MB.R.4430.30; (a) surface scan anterior view, (b) surface scan lateral view; (a1–a4) CT scan slices in parasagittal cross‐section, (b1–b5) CT scan slices in transverse cross‐section. CDF, centrodiapophyseal fossa; dp, diapophysis; nc, neural canal; POCDF, postzygapophyseal centrodiapophyseal fossa; poz, postzygapophysis; pp, parapophysis; prz, prezygapophysis; SPOF, spinopostzygapophyseal fossa; SPRF, spinoprezygapophyseal fossa. Key: Blue highlight, neural canal; green highlight, fossa. Scale bars = 5 cm.

The centra possess apneumatic spongious internal texture. CT scans of anterior (D1–D4, Figures 14 and 15) and middle (D6, D9–D10, Figures 15, 16, 17) dorsal vertebrae reveal that each centrum possesses clustered cavities that do not communicate with external fossae. The clustered cavities in the neural arches of the cervical vertebrae persist into the anterior–middle dorsal vertebrae, with the internal cavities communicating externally via the PRCDF, POCDF and SPOF in D1–D6 (Figures 14, 15, 16). In D9–D10, only the SPOF communicates with internal cavities (Figures 16 and 17). The CDF also communicates with the internal cavities in D1 (MB.R.4404.25), D3 (MB.R.4404.27), D9 (MB.R.4430.30) and D10 (MB.R.4404.34).

#### 3.4.4 Dorsal ribs

Several complete dorsal ribs of ‘Skeleton C’ (MB.R.4430.106–111, MB.R.4430.119–130, MB.R.4430.206–207) and ‘Skeleton 1’ (MB.R.4405.10), and dorsal rib fragments of ‘Skeleton 1’ (MB.R.4405.11) are preserved. Some possess a concavity on the posterior surface between the capitulum and tuberculum (Figure 11g,h), whereas others lack this feature (Figure 11e). Dorsal rib fragments reveal a solid internal texture.

#### 3.4.5 Sacrum

Both ‘Skeleton C’ (MB.R.4430.37, Figure 11l,m) and ‘Skeleton 1’ (MB.R.4405.12–13, MB.R.4405.85, Figure 11n–s) preserve a complete sacrum, each consisting of three vertebrae. These are fused in ‘Skeleton C’, but unfused in ‘Skeleton 1’. The incomplete sacrum of ‘Skeleton 42’ (MB.R.4398.25, Figure 11j,k) preserves two fused vertebrae. The missing sacral vertebra in the latter specimen is inferred to be S3 because the anterior surface of the transverse processes of the anteriormost element is flat and the only other sacral vertebral elements of *Plateosaurus* with this characteristic are S1 in both ‘Skeleton C’ (MB.R.4430.37) and ‘Skeleton 1’ (MB.R.4405.12). An additional isolated element labelled as sacral vertebra 2 (MB.R.4392, Figure 11t,u) is not associated with a skeleton and does not appear to be related to any other elements.

The lateral and ventral surfaces of each sacral centrum are shallowly concave anteroposteriorly, and the lateral surfaces lack a true LPF. In S2 of ‘Skeleton 1’, the lateral surfaces of the centrum each possess a fossa positioned on the dorsal half of the centrum, extending anteroposteriorly. All sacral neural spines possess an SPRF and SPOF. Broken and eroded surfaces on the centra and neural arches reveal a spongious internal texture.

In sacral vertebra 1 of ‘Skeleton C’, the anterior surface of each transverse process is deeply excavated by a prominent fossa immediately lateral to the neural canal. Each fossa is subdivided by a lamina that is orientated ventromedially–dorsolaterally (Figure 11l). The concave posterior surface of the left transverse process of S1 of ‘Skeleton 1’ possesses a small foramen towards its dorsolateral tip, level with the roof of the neural canal (Figure 11o). The anterior surface of each transverse process of S2 of ‘Skeleton 1’ is deeply excavated by a fossa (Figure 11p). However, the features of the posterior surfaces of S1 and the anterior surfaces of S2 of ‘Skeleton C’ and ‘Skeleton 42’ are inaccessible because the sacral vertebrae are fused. The posterior surfaces of each transverse process of S2 of ‘Skeleton 1’ and ‘Skeleton 42’ possess a deep fossa immediately lateral to the neural canal (Figure 11k,q). On the left and right sides of ‘Skeleton 1’, this fossa is subdivided by a prominent lamina orientated ventromedially–dorsolaterally (Figure 11q).

In sacral vertebra 2 of MB.R.4392, the anterior surface of each transverse process is deeply excavated by a prominent fossa immediately lateral to the neural canal, as in S2 of ‘Skeleton 1’. Unlike S2 of ‘Skeleton 1’, however, each fossa of MB.R.4392 is subdivided by a stout lamina that is orientated ventromedially–dorsolaterally (Figure 11t). The posterior surfaces of the transverse processes of MB.R.4392 are transversely flat and dorsoventrally concave (Figure 11u), but incomplete preservation inhibits the assessment of any further characteristics. The anterior surfaces of each transverse process of S3 of ‘Skeleton 1’ possess a deep fossa immediately lateral to the neural canal (Figure 11r), whereas the posterior surfaces are flat, and lack fossae (Figure 11s), as in the posterior surfaces of the transverse processes of S3 of ‘Skeleton C’ (Figure 11m).

#### 3.4.6 Caudal vertebrae

The caudal vertebrae of *Plateosaurus* are represented by Ca1–Ca44 from ‘Skeleton C’ (MB.R.4430.39–80, MB.R.4430.209) and Ca4–Ca39 from ‘Skeleton 1’ (MB.R.4405.15–50). Apart from Ca1, the caudal vertebrae of ‘Skeleton C’ are situated in the correct anatomical position along a metal rod that runs anteroposteriorly through the centra.

The lateral and ventral surfaces of the caudal centra are shallowly concave anteroposteriorly. Two middle caudal vertebrae of ‘Skeleton C’ (Ca13–Ca14, MB.R.4430.50–51) each possess an anteroposteriorly elongate, dorsoventrally short lateral fossa, just ventral to the transverse process on the left side of the centrum (Figure 11v). The anterior–middle caudal vertebrae possess SPRFs and SPOFs that are lost in the posterior caudal vertebrae. Eroded surfaces around the edges of the centra and neural arches reveal a spongious internal texture.

## 4 DISCUSSION

### 4.1 External evidence for PSP in early‐branching sauropodomorphs

#### 4.1.1 Presacral vertebrae

*Thecodontosaurus* lacks external evidence for PSP throughout its axial column, and *Pantydraco* lacks external evidence for PSP in its cervical vertebral series (*contra* Yates, 2003a and Wedel, 2007). In particular, the ‘pseudopleurocoels’ previously described in Ce6–Ce8 of *Pantydraco* (Wedel, 2007; Yates, 2003a) are actually exposed parts of the neural canals. *Ruehleia* possesses external evidence for presacral PSP in the dorsal vertebral sequence only: one subfossa occurs on the left side of each of D9 and D15, and the right side of D10; and two subfossae occur on the right side of D5 and the left side of D13 (see above and Table 3). *Plateosaurus* possesses external evidence for PSP in the posterior cervical vertebrae only: one subfossa in Ce9 of ‘Skeleton 25’ (MB.R.4404.23); and two subfossae in Ce10 of ‘Skeleton C’ (MB.R.4430.21) (see above and Table 3). However, the external pneumatic features of different specimens—and species—of *Plateosaurus* vary (Table 3). In the posterior cervical vertebrae, one adult specimen possesses subdivided PRCDFs (MB.R.4430.21), and three adult specimens possess subdivided POCDFs (MB.R. 4404.23; SMNS F65, Butler et al., 2012; AMNJ 6810, Yates et al., 2012). One adult specimen possesses external evidence for PSP in a middle dorsal vertebra in the form of a subdivided PRCDF (SMNS 12950, Butler et al., 2012). By contrast, a juvenile specimen (MSF 15.8, Nau et al., 2020) and three adult specimens (MB.R.4404; MB.R.4430; SMNS 13200, Schaeffer, 2024) lack external evidence for PSP in all dorsal vertebral elements. This shows that the distribution and extent of PSP differ both inter‐ and intra‐specifically in *Plateosaurus*, as also occurs in extant Aves (Hogg, 1984a, 1984b; Moore & Schachner, 2025; Müller, 1908).

**TABLE 3:** Distribution of external fossae in early‐branching sauropodomorphs. Key: *, subfossa within a fossa (i.e., external evidence for PSP); ^L^, left side; ^R^, right side; –, lack of information.

Taxon	Cervical vertebrae			Dorsal vertebrae			References
	Anterior	Middle	Posterior	Anterior	Middle	Posterior	
*Buriolestes schultzi*	0	0	0	PRCDF CDF POCDF	CDF POCDF	CDF POCDF	Müller et al. (2018)
*Pampadromaeus barberenai*	–	–	–	PRCDF CDF POCDF	PRCDF CDF POCDF	PRCDF CDF POCDF	Langer et al. (2019)
*Pantydraco caducus* (juvenile)	SPRF	SPRF	SPRF PRCDF CDF POCDF SPOF	–	–	–	This study
*Thecodontosaurus antiquus*	SPRF SPOF	SPRF SPOF	SPRF POCDF SPOF	–	SPRF PRCDF CDF POCDF SPOF	SPRF CDF POCDF SPOF	Ballell et al. (2020), This study
*Ruehleia bedheimensis*	SPRF SPOF	SPRF PRCDF CDF SPOF	SPRF PRCDF CDF POCDF SPOF	SPRF PRCDF*^R^ CDF*^R^ POCDF SPOF	SPRF PRCDF CDF*^R^ POCDF*^L^ SPOF	SPRF PACPRF CDF*^L^ POCDF*^L^ SPOF	This study
*Macrocollum itaquii* (subadult paratype)	–	–	SPRF CDF CPOF SPOF	PRCDF*^R^ CPRF CDF CPOF POCDF	–	–	Aureliano, Ghilardi, et al. (2024)
*Plateosaurus* sp. SMNS F65, 12950	–	–	PRCDF CDF POCDF*^R^	–	PRCDF*^R^ CDF POCDF	–	Butler et al. (2012)
*Plateosaurus engelhardti* AMNH 6810	–	–	PRCDF CDF POCDF*^LR^	CDF POCDF	–	–	Yates et al. (2012)
*Plateosaurus longiceps* (MB.R.4404, 4430)	SPRF SPOF	SPRF CDF SPOF	SPRF PRCDF*^LR^ CDF POCDF*^R^ SPOF	SPRF PRCDF CDF POCDF SPOF	SPRF PRCDF CDF POCDF SPOF	SPRF CDF POCDF SPOF	This study
*Plateosaurus trossingensis* SMNS 13200 (adult holotype)	–	–	–	PRCDF CDF POCDF	PRCDF CDF POCDF	CDF POCDF	Schaeffer (2024)
*Plateosaurus trossingensis* MSF 15.8 (juvenile referred specimen)	SPRF SPOF	SPRF	PRCDF CDF POCDF	SPRF PRCDF CDF POCDF	PRPADF PACDF POCDF	PACDF POCDF SPOF	Nau et al. (2020)
*Eucnemesaurus entaxonis*	–	–	–	–	–	CDF	McPhee et al. (2015)
*Eucnemesaurus fortis*	–	–	–	–	CDF*^R^ POCDF	CDF POCDF*^R^	Yates (2007); Yates et al. (2012)
*Riojasaurus incertus*	0	–	PRCDF	PRCDF CDF	–	–	Bonaparte (1999)
*Kholumolumo ellenbergerorum*	–	–	SPRF PRCDF CDF POCDF SPOF	SPRF PRCDF CDF POCDF SPOF	–	–	Peyre de Fabrègues and Allain (2020)
*Sarahsaurus aurifontanalis*	SPRF SPOF	SPRF SPOF	SPRF SPOF	PRCDF CDF POCDF	PRCDF CDF POCDF	–	Marsh and Rowe (2018)
*Massospondylus carinatus*	–	SPRF SPOF	SPRF SPOF	SPRF PRPADF CDF SPOF	SPRF PRPADF CDF POCDF SPOF	SPRF PRPADF CDF POCDF SPOF	Barrett et al. (2019)
*Seitaad ruessi*	–	–	–	–	PRCDF CDF POCDF	CDF POCDF	Sertich and Loewen (2010)
*Xingxiulong chengi*	–	–	–	–	PRCDF CDF POCDF	–	Wang et al. (2019)
*Aardonyx celestae* (immature)	–	–	–	–	–	CDF POCDF*^R^	Yates et al. (2012)
*Irisosaurus yimenensis*	0	0	POCDF SPOF	CDF	–	–	Peyre de Fabrègues et al. (2020)
*Sefapanosaurus zastronensis*	SPRF SPOF	–	–	–	PRCDF CDF POCDF	CDF POCDF	Otero et al. (2015)
*Melanorosaurus readi*	–	SPRF	–	PRCDF^*L^ CDF POCDF SPOF	SPRF PRCDF CDF POCDF SPOF	–	Barrett and Choiniere (2024)
*Camelotia borealis*	–	–	–	–	–	SPRF CDF POCDF*^L^ SPOF	Yates et al. (2012), SLB, personal observation
*Pulanesaura eocollum*	SPRF SPOF	–	–	SPRF PRCDF POCDF SPOF	SPRF PRCDF CDF POCDF SPOF	POCDF*^LR^ SPOF	Yates et al. (2012), McPhee and Choiniere (2018)
*Antetonitrus ingenipes* (immature)	–	–	–	SPRF POCDF SPOF	SPRF CDF POCDF*^LR^ SPOF	SPOF	Yates et al. (2012), McPhee et al. (2014)
*Ingentia prima*	–	PRCDF CDF	PRCDF*^R^ CDF*^R^	–	–	–	Apaldetti et al. (2018)
*Lessemsaurus sauropoides*	–	–	PRCDF CDF	PRCDF CDF POCDF	–	–	Apaldetti et al. (2018)
*Schleitheimia schutzi*	–	–	–	PRCDF CDF POCDF	–	–	Rauhut et al. (2020)

**FIGURE 18:**
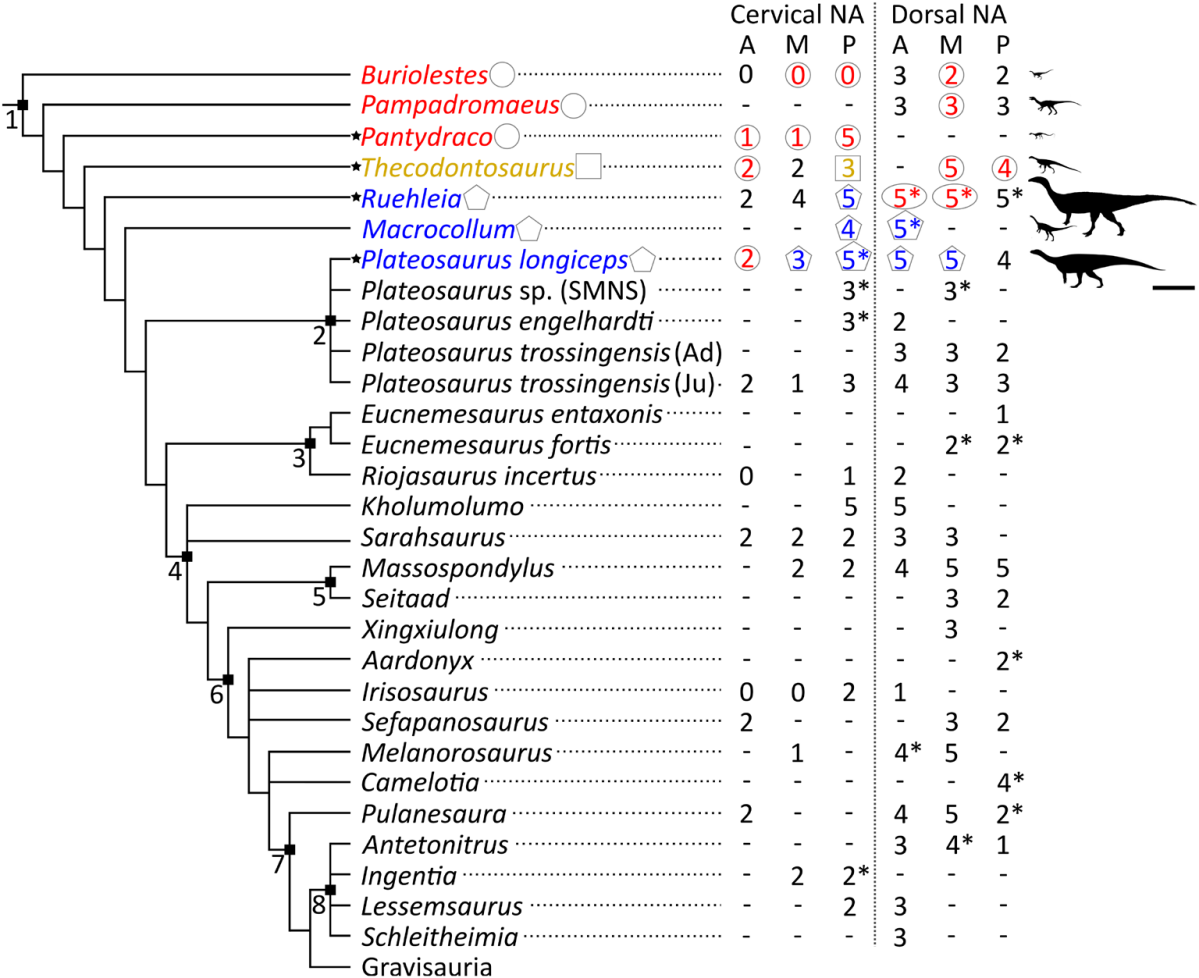
Cladogram of early‐branching sauropodomorphs with neural arch fossae count in presacral vertebrae based on information from Table 3 and the distribution of external and internal PSP. Phylogenetic positions of included taxa are based on the analyses of Müller et al. (2018), Peyre de Fabrègues and Allain (2020), Beccari et al. (2021), Gomez et al. (2021) and Schaeffer (2024). Silhouettes taken from https://www.phylopic.org (*Pampadromaeus barberenai*, *Plateosaurus longiceps*, *Ruehleia bedheimensis*, *Thecodontosaurus antiquus*) and modified from Yates (2003a: *Pantydraco caducus*), Müller et al. (2018: *Buriolestes schultzi*) and Bem & Müller (2024: *Macrocollum itaquii*). A, anterior; Ad, adult; Ju, juvenile; M, middle; NA, neural arch; P, posterior. Key: Black colour and no shape, internal PSP data unavailable; black star, taxa CT scanned in this study; blue colour and pentagon shape, internal PSP present; red colour and circle shape, internal PSP absent; yellow colour and square shape, internal PSP possibly present; *, external evidence for PSP; –, lack of information. Clades: 1, Sauropodomorpha; 2, Plateosauridae; 3, Riojasauridae; 4, Massopoda; 5, Massospondylidae; 6, Sauropodiformes; 7, Sauropoda; 8, Lessemsauridae. Scale bar = 2 m.

In addition to *Plateosaurus*, and newly identified in *Ruehleia*, external evidence for presacral PSP has previously been claimed in several other early‐branching sauropodomorphs (Table 3). These include *Lessemsaurus sauropoides* from the Late Triassic of Argentina, in which external evidence for PSP was noted on the posterior cervical and anterior dorsal vertebrae (Apaldetti et al., 2018). However, we disagree with this interpretation: Ce8 possesses a PRCDF and CDF, and D2–D3 possess a PRCDF, CDF and POCDF (Apaldetti et al., 2018: fig. S4); however, none of these fossae are deep, and nor are they subdivided (*contra* Apaldetti et al., 2018), and thus, they do not represent unambiguous evidence for PSP. As such, we restrict evidence for external PSP in the presacral vertebrae to the following early‐branching sauropodomorphs: the posterior cervical vertebrae of *Ingentia prima* (Apaldetti et al., 2018); the posterior cervical and middle dorsal vertebrae of *Plateosaurus* (Butler et al., 2012; Yates et al., 2012; This study); the anterior–posterior dorsal vertebrae of *Ruehleia* (This study); the anterior dorsal vertebrae of *Macrocollum taquii* (Aureliano, Ghilardi, et al., 2024) and *Melanorosaurus readi* (Barrett & Choiniere, 2024); the middle dorsal vertebrae of *Antetonitrus ingenipes* (McPhee et al., 2014; Yates et al., 2012); the middle–posterior dorsal vertebrae of *Eucnemesaurus fortis* (Yates et al., 2012); and the posterior dorsal vertebrae of *Aardonyx celestae* (Yates et al., 2012), *Camelotia borealis* (Yates et al., 2012; SLB, personal observation) and *Pulanesaura eocollum* (Yates et al., 2012; McPhee & Choiniere, 2018; Table 3, Figure 18). In each of these species, neural arch fossae possess foramina and/or are subdivided, and subdivision occurs within the PRCDF, CDF and/or POCDF (Table 3). Furthermore, there is a general trend within an individual for external features of PSP to occur asymmetrically on only one side of an element and on only one element within a sequence. Our new data, combined with a critical appraisal of the literature, suggest that the distribution of external evidence for PSP in earlier branching sauropodomorphs does not follow a common pattern across the axial column (Table 3, Figure 18).

#### 4.1.2 Sacral and caudal vertebrae

The concave posterior surfaces of the transverse processes of sacral vertebra 1 of *Ruehleia* and S1–S2 of *Plateosaurus* (‘Skeleton 1’) possess external evidence for PSP: a small foramen is located within a fossa on the left side of S1 of *Plateosaurus*; and there is a subdivided fossa in the same position on the right side of S1 of *Ruehleia* and the left side of S2 of *Plateosaurus*. Posterior to the transverse processes, the lateral surfaces of the centrum of S2 of *Plateosaurus* (‘Skeleton 1’) each possess a fossa.

Prior to this study, external evidence for sacral PSP had been identified in three early‐branching sauropodomorphs: *Pampadromaeus*; *Aardonyx*; and *Anchisaurus polyzelus* (Langer et al., 2019; Yates, 2004; Yates et al., 2012). Langer et al. (2019) described the left side of S1 of *Pampadromaeus* as possessing an anteroposteriorly elongate furrow just posterior to the parapophysis and ventral to the neurocentral junction. From observation of photographs (J. Lovegrove, personal communication, 2025), this lateral fossa presents only ambiguous evidence for PSP because a connection with internal chambers cannot be assessed. S1 of *Aardonyx* possesses a fossa on each lateral surface of the centrum, and a subfossa is located on the concave posterior surface of the left transverse process (Yates et al., 2012: fig. 8). S1 of *Anchisaurus* possesses a foramen that opens ventrally at the base of the sacral rib (Yates, 2004: fig. 1). Currently, no external evidence for PSP has been described or identified in the caudal vertebrae of early‐branching sauropodomorphs.

### 4.2 Internal evidence for PSP in early‐branching sauropodomorphs

The cervical vertebrae of *Pantydraco* are apneumatic (Figure 7). *Thecodontosaurus* possesses ambiguous evidence for internal pneumaticity in the posterior cervical vertebrae: BRSMG Cb 4155 possesses less densely packed trabeculae dorsomedial to each POCDF, approximately where the SPOF would be expected to communicate internally. The two cavities located ventrolateral to the SPOF in BRSMG Cb 4167 potentially represent paired camerate cavities, approximately where the POCDF would be expected to communicate internally (Figure 1c,d). If correct, this communication between external fossae and internal cavities would be indicative of PSP. However, we urge caution when evaluating the PSP of an element from a broken cross‐section. The middle–posterior dorsal vertebrae and anterior–middle caudal vertebrae of *Thecodontosaurus* lack internal evidence for PSP, as revealed by CT scans (Figures 2, 3, 4). This agrees with Benton et al. (2000), who described the posterior dorsal vertebrae of *Thecodontosaurus* as possessing a highly ‘cancellous’ internal texture with a virtually hollow centrum, as revealed from broken cross‐sections.

**FIGURE 19:**
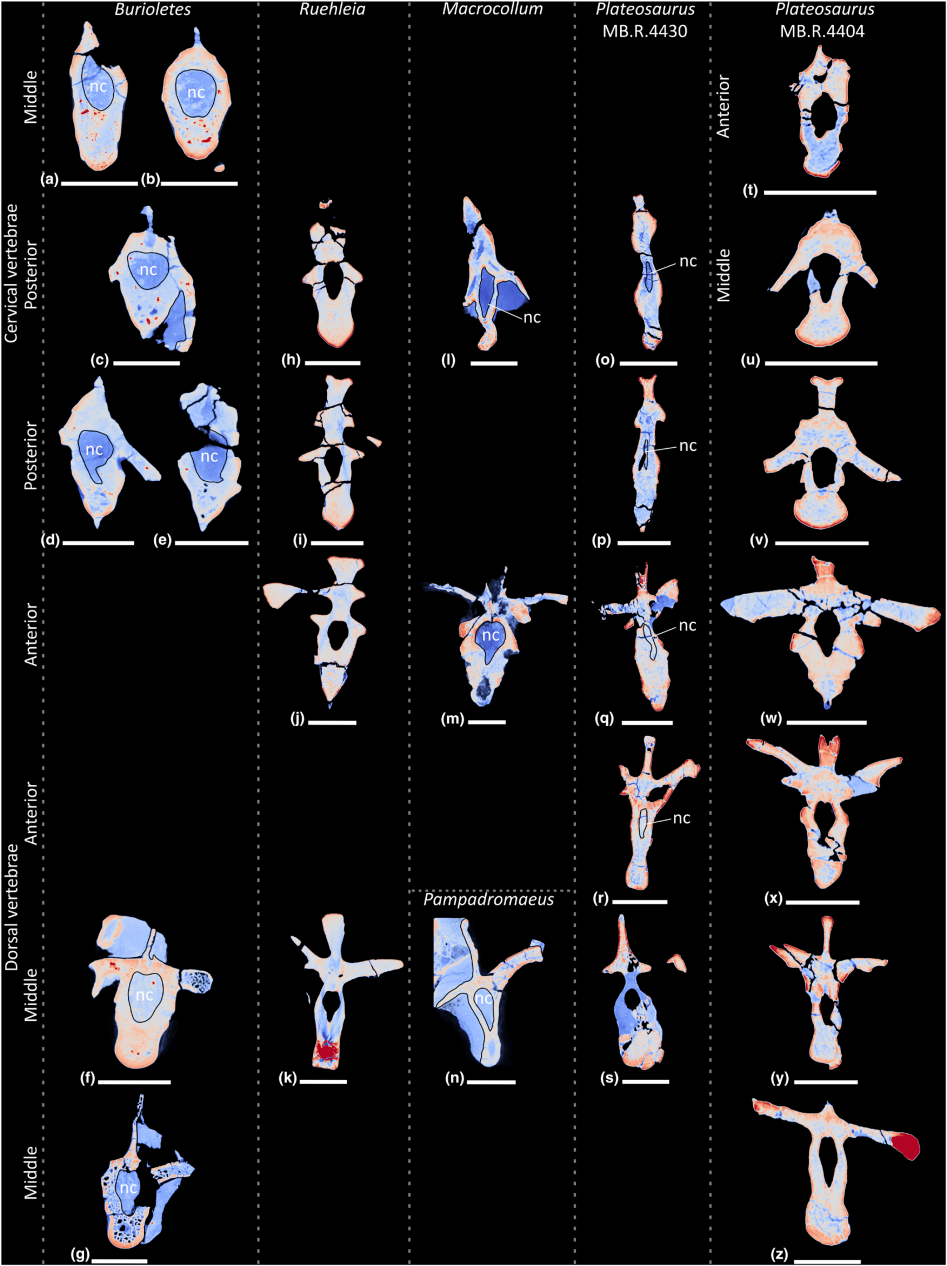
Colour density map outlining PSP in CT scan slices in transverse cross‐section of the presacral vertebrae of early‐branching sauropodomorphs. (a–g) *Buriolestes schultzi*, (h–k) *Ruehleia bedheimensis*, (l–m) *Macrocollum itaquii*, (n) *Pampadromaeus barberenai*, (o–z) *Plateosaurus longiceps*; (t) anterior cervical vertebra, (a–b, u) middle cervical vertebrae, (c–e, h–i, l, o–p, v) posterior cervical vertebrae, (j, m, q–r, w–x) anterior dorsal vertebrae, (f–g, k, n, s, y–z) middle dorsal vertebrae. nc, neural canal. Scale bars = 2 cm (*Buriolestes*, *Macrocollum*, *Pampadromaeus*), 5 cm (*Ruehleia*, *Plateosaurus*).

**FIGURE 20:**
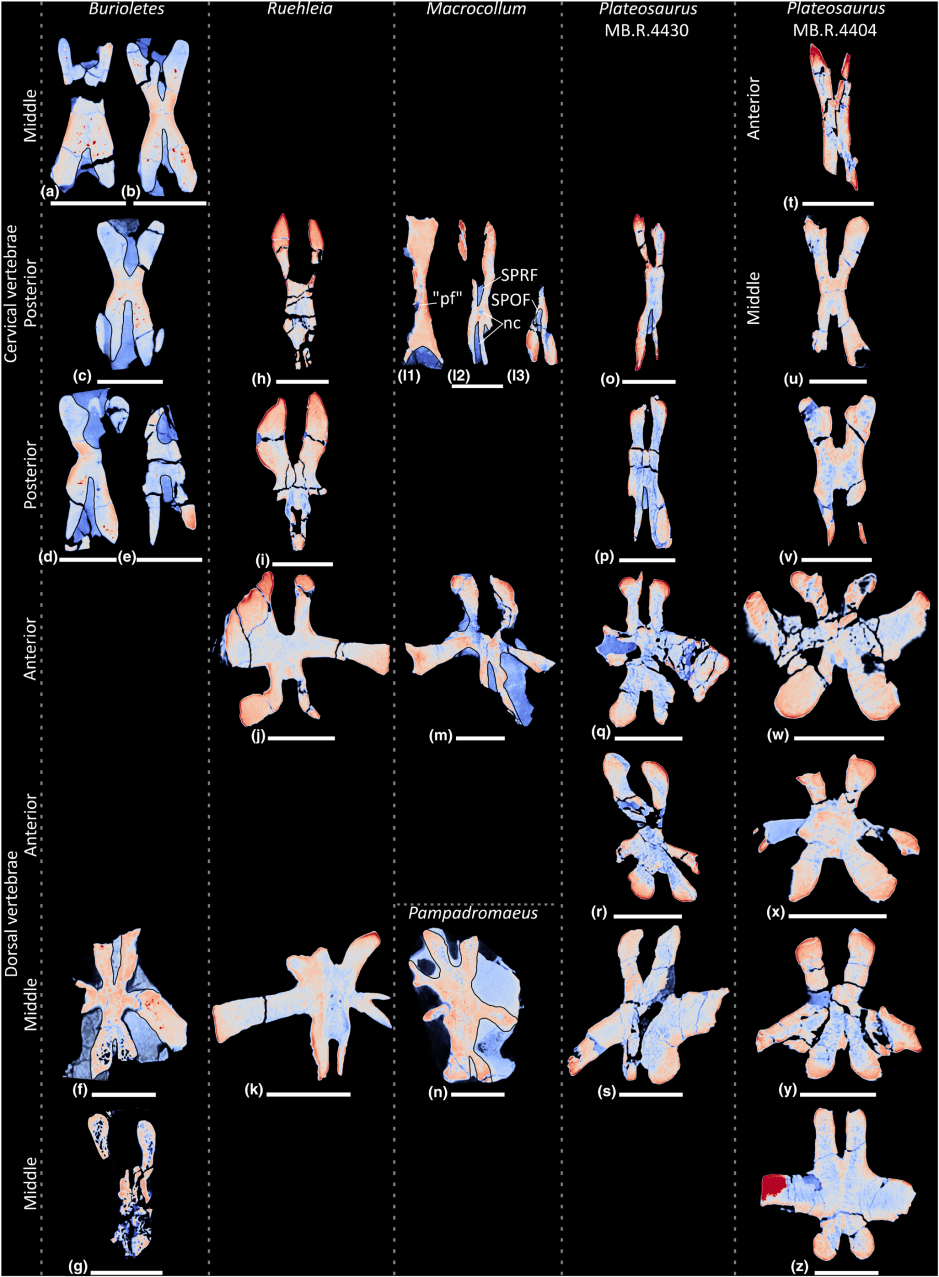
Colour density map outlining PSP in CT scan slices in horizontal cross‐section of the presacral vertebrae of early‐branching sauropodomorphs. (a–g) *Buriolestes schultzi*, (h–k) *Ruehleia bedheimensis*, (l–m) *Macrocollum itaquii*, (n) *Pampadromaeus barberenai*, (o–z) *Plateosaurus longiceps*; (t) anterior cervical vertebra, (a–b, u) middle cervical vertebrae, (c–e, h–i, l, o–p, v) posterior cervical vertebrae, (j, m, q–r, w–x) anterior dorsal vertebrae, (f–g, k, n, s, y–z) middle dorsal vertebrae; (l1) CT scan slice through centrum, (l2) CT scan slice through neural arch, (l3) CT scan slice through neural spine. nc, neural canal; ‘pf’, pneumatic foramen; SPOF, spinopostzygapophyseal fossa; SPRF, spinoprezygapophyseal fossa. Scale bars = 2 cm (*Buriolestes*, *Macrocollum*, *Pampadromaeus*), 5 cm (*Ruehleia*, *Plateosaurus*).

The clustered cavities located within the cervical and dorsal centra of *Ruehleia* are surrounded by a thick, dense layer of bone, and they do not communicate with an LPF; they are therefore interpreted as apneumatic cavities (Figure 19h–k). In the posterior cervical vertebrae of *Ruehleia* (Ce9–Ce10), the SPOF communicates with small internal cavities, therefore possessing unambiguous evidence for PSP (Figure 20h,i). These internal cavities are neither exclusively camerate nor camellate, and they do not align with the ‘protocamerate’ bone structure proposed by Aureliano, Ghilardi, et al. (2024). However, we refrain from introducing a new pneumatic term at this time. Internal pneumaticity does not continue into the dorsal vertebrae of *Ruehleia*, with the centra and neural arches of D1 and D5 lacking communication between the internal cavities and external fossae (Figure 20j,k). We therefore interpret the dorsal vertebral fossae to be incipient pneumatic characteristics, with the subdivided fossae described above representing an early form of pneumatic diverticulum that eventually extended from the posterior cervical vertebrae to invade the dorsal vertebral column. The development of pneumatic diverticula might not have been sequential, given that D1 lacks subfossae and the internal texture within the centrum and neural arches is the same, unlike more posteriorly placed dorsal vertebral elements.

The centra of the anterior–posterior cervical vertebrae of *Plateosaurus* possess apneumatic clustered cavities (Figure 19o,p,t–v), as described above for *Ruehleia*. The neural arches of the cervical vertebrae possess small internal cavities. They do not communicate externally in Ce4 (Figure 20t), meaning they must be apneumatic. However, the internal cavities within the middle–posterior cervical neural arches communicate externally with the CDF and the SPOF, and with the subdivided right POCDF in Ce9 of ‘Skeleton 25’, providing unambiguous evidence for PSP (Figures 19o,p,u,v and 20o,p,u,v). The subdivided left and right PRCDF on Ce10 of ‘Skeleton C’ do not communicate with any internal cavities (Figure 13c2,c4). This suggests that caution is needed when inferring internal pneumaticity purely from external fossae alone. The internal pneumatic cavities in the cervical neural arches of *Plateosaurus* resemble those seen in *Ruehleia*; they do not align with the definitions for camerae, camellae or protocamerae, but are unambiguous evidence for PSP.

In the anterior and proximal middle dorsal vertebrae (D1–D4 and D6) of *Plateosaurus* (Figures 19q–r,w–y and 20q–r,w–y), an increase in PSP occurs relative to the cervical vertebrae, with the internal cavities communicating externally via three fossae (PRCDF, POCDF and SPOF) in all elements, and an additional fossa (CDF) in D1 and D3. In the distal middle dorsal vertebrae, D9–D10 (Figures 19s,z and 20s,z), a decrease in PSP occurs, with the internal cavities communicating externally via only two fossae (CDF and SPOF). Therefore, in *Plateosaurus*, PSP extends from the middle cervical vertebrae to at least the middle dorsal vertebrae. PSP differs between *Plateosaurus* specimens examined and does not develop in a sequential pattern, as evidenced above by the difference in external fossa/internal cavity communication observed D1–D4. Unfortunately, no posterior dorsal vertebrae of *Plateosaurus* were CT scanned.

**FIGURE 21:**
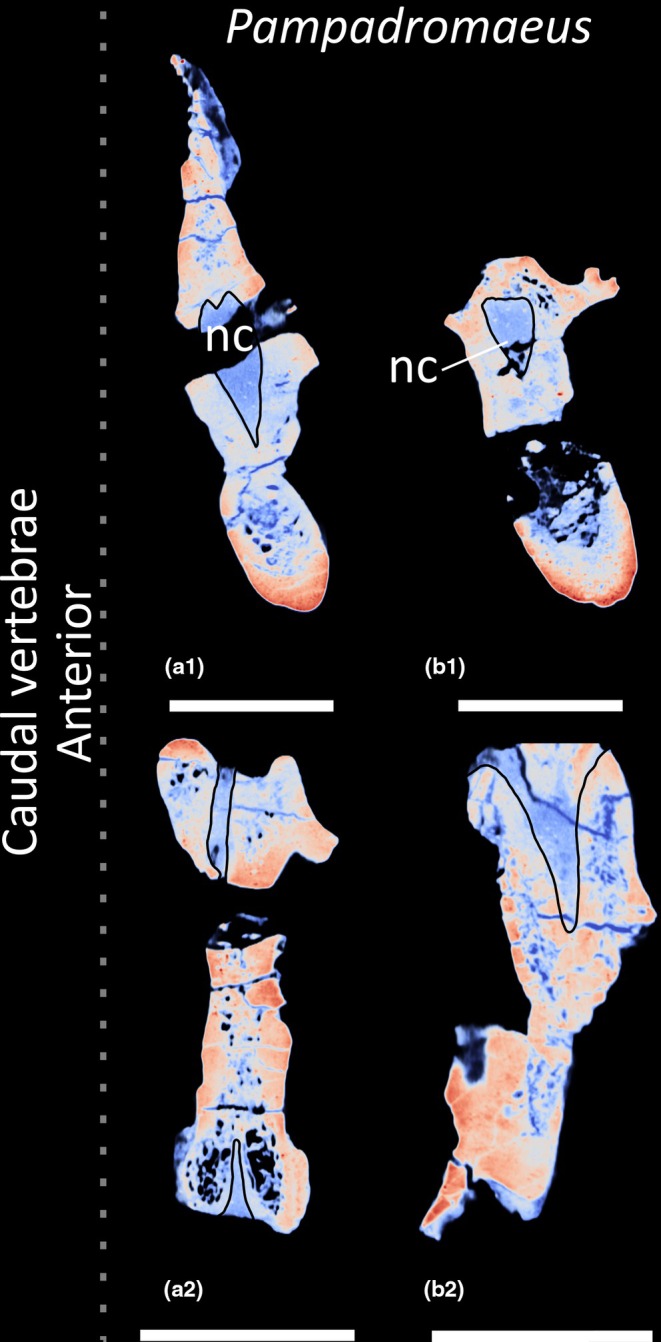
Colour density map outlining PSP in CT scan slices of the anterior caudal vertebrae of *Pampadromaeus barberenai*. (a1, b1) Transverse cross‐section, (a2, b2) horizontal cross‐section. nc, neural canal. Scale bars = 2 cm.

CT scans of presacral vertebrae are available for three additional early‐branching sauropodomorphs, *Buriolestes*, *Pampadromaeus* and *Macrocollum* (Figures 19, 20, 21; Aureliano et al., 2022; Aureliano, Ghilardi, et al., 2024). In *Buriolestes*, the middle–posterior cervical vertebrae (Ce5–Ce9, Figures 19a–e and 20a–e) and middle dorsal vertebrae (D8–D9, Figures 19f,g and 20f,g) possess apneumatic chaotic trabeculae in the centra and neural arches, as is also the case for the middle dorsal vertebra of *Pampadromaeus* (Figures 19n and 20n; Aureliano et al., 2022). Aureliano et al. (2022) also described two supposed anterior dorsal vertebrae of *Pampadromaeus* as possessing a ‘new type’ of apneumatic internal texture in the centra, which they termed ‘pseudo‐polycamerate’. However, these elements are actually anterior caudal vertebrae (Figure 21; see also Langer et al., 2019: fig. 19a). This ‘pseudo‐polycamerate’ texture (Figure 21a1,b1) appears to be essentially the same as the apneumatic chaotic trabecular pattern described for the neural arches of those elements (Figure 21a2,b2; Aureliano et al., 2022), as well as the middle dorsal vertebrae of *Buriolestes* (Figures 19f,g and 20f,g; *contra* Aureliano et al., 2022, p. 8). Based on our observation of cross‐sections of the CT data (Figures 19, 20, 21), we do not recognise a unique internal texture (i.e., ‘pseudo‐polycamerae’) in the CT scanned vertebrae of *Pampadromaeus*.

The posterior cervical centrum of *Macrocollum* possesses dense trabecular bone with a narrow, elongated chamber connecting two external concavities that were interpreted as LPF by Aureliano, Ghilardi, et al. (2024). However, observation of the CT scans of this element reveals that the lateral surfaces of the centrum do not possess true LPF (Figures 19l and 20l1) and the ‘pneumatic foramen’ (sensu Aureliano, Ghilardi, et al., 2024: fig. 2) represents an anteroposteriorly concave lateral surface that lacks a true LPF. Furthermore, the supposed chamber within the centrum that communicates externally with the ‘pneumatic foramen’ appears to be broken external bone that has deformed to create a superficial internal cavity (Figure 20l1), and the internal texture of the centrum is composed of densely packed trabeculae (Figures 19l and 20l1). Therefore, there is no communication between external fossae and an internal chamber, refuting the presence of unambiguous PSP in the centrum of this element. Rather, the posterior cervical and anterior dorsal centra of *Macrocollum* possess chaotic apneumatic trabeculae. By contrast, the neural arches of the posterior cervical and anterior dorsal vertebrae of *Macrocollum* do possess evidence for unambiguous PSP, including a decrease in trabecular density in comparison to their respective centra, and the presence of several external fossae that communicate with internal chambers (Figures 19l–m and 20l2,l3,m). Aureliano, Ghilardi, et al. (2024) proposed a new type of pneumatic bone structure for the neural arches of these elements, termed ‘protocamerate’, as an intermediate type of skeletal pneumaticity that possesses both camerate and camellate chambers. However, we disagree with this new term being applied to both elements. The neural arch of the posterior cervical vertebra possesses distinct, separate internal chambers that communicate externally via the CDF, POCDF, SPRF and SPOF (Figure 19l). These chambers do not bifurcate, communicate with each other, nor possess a camellate array (*contra* Aureliano, Ghilardi, et al., 2024), and the bone around these chambers is solid (Figures 19l and 20l2–l3). By contrast, the neural arch of the anterior dorsal vertebra possesses internal chambers that align with the ‘protocamerate’ definition in being neither exclusively camerate nor camellate, and they communicate externally via the same fossae as the posterior cervical vertebra (Figures 19m and 20m).

We urge caution in defining new apneumatic and pneumatic bone structures in fossil taxa based purely on qualitative metrics until the characteristics of PSP are better constrained and understood. The current terminology used to describe internal pneumaticity was established for true sauropods and may not be applicable to PSP in early‐branching sauropodomorphs. Instead, we stress the need for a more quantitative framework to be established in the future that includes a better differentiation between pneumatic and apneumatic internal cavities.

Thus far, sacral vertebrae of early‐branching sauropodomorphs have not been CT scanned, limiting the interpretation of external features as pneumatic. However, it is interesting that three taxa (*Ruehleia*, *Plateosaurus*, and *Aardonyx*) possess a subfossa in essentially the same position on the transverse process. Given that PSP in the presacral vertebrae of early‐branching sauropodomorphs appears to manifest first in the neural arches, it is possible that the neural arches of these sacral vertebrae could have been pneumatised via these subfossae. There is no evidence for internal PSP in the caudal vertebrae of those early‐branching sauropodomorphs that have been CT scanned, that is, *Pampadromaeus* and *Thecodontosaurus*.

### 4.3 Implications of newly described PSP in Late Triassic sauropodomorphs

From this survey of seven early‐branching sauropodomorphs that have had their presacral vertebrae CT scanned, the presence of PSP appears to exhibit a phylogenetic signal, with PSP more prevalent in more deeply nested taxa, that is, those closer to the sauropod radiation (Figure 18). Unambiguous evidence for PSP occurs in the neural arches of the middle cervical and dorsal vertebrae of one taxon (*Plateosaurus*), the posterior cervical vertebrae of three taxa (*Ruehleia*, *Macrocollum* and *Plateosaurus*) and the anterior dorsal vertebrae of two taxa (*Macrocollum* and *Plateosaurus*). Pneumatisation occurs via fossae of the neural arches, and there is no evidence for pneumatisation via paramedullary (neural canal) diverticula. However, osteological correlates of paramedullary diverticula are not always visible in extant Aves (Atterholt & Wedel, 2023). The apneumatic cavities in the centra and the pneumatic cavities in the neural arches of the sampled sauropodomorph taxa do not connect with the neural canal. Consequently, if paramedullary diverticula existed in early‐branching sauropodomorphs, they either did not leave osteological correlates or they did not invade the presacral column. *Macrocollum* no longer represents the only non‐gravisaurian sauropodomorph to possess cervical and anterior dorsal vertebral pneumatisation, because *Plateosaurus* also possesses these features (see above). We hypothesise that early‐branching sauropodomorphs more derived than *Macrocollum* and *Plateosaurus* (i.e., massopodans; Figure 18) will also possess this pneumatisation pattern, but this is speculative in the absence of CT data.

In early‐branching sauropodomorphs, PSP probably manifested itself first in the neural arches of the posterior cervical vertebrae and then progressed from this point anteriorly and/or posteriorly along the vertebral column, with the extent of PSP decreasing away from its point of origin (Wedel, 2006). CT data reveal that the lagerpetid *Venetoraptor gassenae* possesses PSP in the neural arches of the anterior dorsal vertebrae but that the centra of the anterior cervical and dorsal vertebrae lack PSP (Aureliano et al., 2025). Early‐branching pterosaurs possess reduced PSP in comparison to their later branching descendants, with PSP restricted to posterior cervical and dorsal vertebrae in the former group (Claessens et al., 2009). In early‐branching theropods, PSP manifests first in the postaxial cervical neural arches and the cervical to anterior dorsal centra (Benson et al., 2012). In later branching theropods, PSP extends from this point of origin anteriorly via a ‘centrum‐first’ pattern and posteriorly via a ‘neural arch‐first’ pattern (Benson et al., 2012). In extant birds, the ontogenetic development of PSP varies among species, with axial pneumatisation occurring at different points of origin, and in anterior‐to‐posterior or posterior‐to‐anterior directions, depending on the species (Moore & Schachner, 2025). Future studies on the distribution of PSP in the axial column of non‐eusauropod sauropodomorphs should also assess whether serial variation may correspond to differences in invasion from the cervical versus abdominal air sacs.

Occurrences of PSP in Late Triassic early‐branching sauropodomorphs do not appear to co‐occur consistently with increases in body mass (Table 4, Figure 18). Instead, PSP increases in extent in later branching taxa (Figure 18). *Buriolestes*, *Pampadromaeus* and *Macrocollum* are all approximately the same body mass (Table 4) and yet of the three taxa, only the latter possesses PSP. *Buriolestes* and *Pampadromaeus* are almost double the mass of *Thecodontosaurus* (Table 4), but the latter is the only one of these taxa to potentially possess PSP. *Ruehleia* is marginally heavier than *Plateosaurus* (1140 vs. 917 kg, respectively) but exhibits less extensive PSP than the latter, and PSP occurs throughout more of the vertebral column of *Macrocollum* than *Ruehleia*, despite *Macrocollum* being only 8.7 kg. Contemporaneous sauropodomorphs that are closer to the sauropod radiation are much heavier, with *Lessemsaurus sauropoides* weighing between 5 and 7 tonnes (Apaldetti et al., 2018), yet lacking external evidence for PSP in the posterior cervical and anterior dorsal vertebrae. Conversely, pterosaurs appear to possess a strong correlation between the extent of pneumaticity and body size (Claessens et al., 2009), and theropods possess a weak but statistically significant correlation between pneumaticity and femur length (Benson et al., 2012). However, small‐bodied pterosauromorphs and theropods still possess PSP (Aureliano et al., 2025; Benson et al., 2012; Claessens et al., 2009). In a study on 100 species of extant anseriform birds, O'Connor (2004) did not find any significant correlation between pneumaticity and body size. Furthermore, the skeletons of more massive birds are heavier than those of smaller birds, irrespective of pneumaticity status (Moore & Schachner, 2025). This means that when assessing the development of PSP in avemetatarsalians, an increase in size alone cannot be regarded as the only driving force. Rather, the development of PSP—and therefore an avian‐style respiratory system—in early‐branching sauropodomorphs is perhaps better linked with its possible physiological effects, such as lightening the neck, an increased ability to shed excess heat and more efficient oxygen uptake at a time when atmospheric O_2_ levels were low (Berner et al., 2007; Sander, 2013). Once developed, PSP could have then been secondarily exapted for gigantism in sauropods (Sander, 2013).

**TABLE 4:** Body mass estimates and development of PSP in early‐branching sauropodomorphs that have been CT scanned.

Taxon	Body mass (kg)	References	PSP
*Buriolestes schultzi*	7	Moro et al. (2024)	Absent
*Pampadromaeus barberenai*	8.5	Benson et al. (2014)	Absent
*Pantydraco caducus*	1.2	Benson et al. (2014)	Absent
*Thecodontosaurus antiquus*	4.4	This study	PSP in posterior cervical vertebrae
*Ruehleia bedheimensis*	1140	McPhee et al. (2018)	PSP in posterior cervical vertebrae
*Macrocollum itaquii*	8.7	Apaldetti et al. (2021)	PSP in posterior cervical and anterior dorsal vertebrae
*Plateosaurus longiceps*	917	McPhee et al. (2018)	PSP in middle cervical–middle dorsal vertebrae

*Note*: The body mass of *Thecodontosaurus antiquus* was calculated using the formula of Benson et al. (2014) and the femoral circumference measurement from Moro et al. (2024: tab. S1).

### 4.4 Did PSP evolve once or multiple times?

Within Archosauria, PSP is absent in pseudosuchian and non‐archosaurian archosauromorphs (Britt, 1993; Butler et al., 2012; Dalle‐Laste et al., 2025; O'Connor, 2006). Within Avemetatarsalia, PSP is absent in *Mambachiton andohana*, aphanosaurs, *Lagosuchus talampayensis*, silesaurids, non‐dinosaurian dinosauromorphs and ornithischians, but present in pterosauromorphs, theropods and sauropodomorphs (Agnolin & Ezcurra, 2019; Aureliano et al., 2025; Benson et al., 2012; Britt, 1993; Butler et al., 2009, 2012; Nesbitt et al., 2023; O'Connor, 2006; Wedel, 2006). This raises the question of when PSP developed, and whether it evolved once at the base of Avemetatarsalia and was secondarily lost in multiple clades, or if it independently evolved at least three times in pterosauromorphs, theropods and sauropodomorphs. Internal evidence for PSP was recently reported in the anterior dorsal neural arches of the Late Triassic (Carnian)‐aged lagerpetid *Venetoraptor gassenae* (Aureliano et al., 2025), but it has not been reported in the contemporaneous *Ixalerpeton polesinensis* (Cabreira et al., 2016). Unfortunately, axial material of Norian‐aged lagerpetids is currently unknown (Butler et al., 2012; Garcia & Müller, 2025). PSP has been recognised in the early‐branching pterosaurs *Raeticodactylus filisurensis* and *Austriadraco dallavecchiai* from the Late Triassic (Norian), as well as the dimorphodontid *Dimorphodon macronyx* from the Early Jurassic Hettangian–Sinemurian (Butler et al., 2009; Kellner, 2015). Based on external evidence, PSP occurs in an unidentified cervical centrum of *Dimorphodon*, the middle cervical neural arches of *Austriadraco* and *Raeticodactylus*, the anterior dorsal centra of *Raeticodactylus* and dorsal centra D5–D9 of *Dimorphodon* (Butler et al., 2009). In the Late Triassic (Carnian) herrerasaurian saurischians (Baron et al., 2017), external evidence for PSP is absent in *Herrerasaurus ischigualastensis* (Benson et al., 2012), and *Gnathovorax cabreirai* lacks external and internal evidence for PSP (Aureliano et al., 2022). External evidence for PSP has been reported in the Norian‐aged non‐neotheropods *Eodromaeus murphi* (Martínez et al., 2011) and *Tawa hallae* (Nesbitt et al., 2009), and the Norian–Rhaetian‐aged neotheropod *Coelophysis bauri* (Colbert, 1989). PSP occurs in the anterior–middle cervical centra of *Tawa*, the anterior–middle cervical neural arches of *Coelophysis* and the posterior cervical centra of *Eodromaeus* (Benson et al., 2012; Colbert, 1989; Martínez et al., 2011; Nesbitt et al., 2009). By the Early Jurassic, PSP extends across more of the axial skeleton of neotheropods, with *Dilophosaurus wetherilli* possessing external evidence for PSP in the postaxial cervical centra and neural arches, and the anterior dorsal centra (Britt, 1993). PSP is absent in the Carnian‐aged early‐branching sauropodomorphs *Buriolestes* and *Pampadromaeus* (see above). PSP is present in the Norian‐aged *Macrocollum*, *Ruehleia* and *Plateosaurus* (see above) and might be present in the Rhaetian‐aged *Thecodontosaurus* (see above).

From this survey, PSP appears to have been absent in Carnian‐aged saurischians, but present in multiple taxa in both theropod and sauropodomorph clades by the Norian. At least one species of Carnian‐aged lagerpetid—early‐branching pterosauromorphs outside of the pterosaur radiation (Ezcurra et al., 2020; Garcia & Müller, 2025)—possesses PSP (Aureliano et al., 2025). True pterosaurs first appear in the fossil record only in the Norian, and all early members of the clade possess PSP (Aureliano et al., 2025; Butler et al., 2009). This supports the idea that PSP evolved at least three times in Avemetatarsalia in pterosauromorphs, theropods and sauropodomorphs.

## 5 CONCLUSIONS

Several conclusions can be drawn from our new observations and critical appraisal of the literature. In early‐branching Late Triassic sauropodomorphs, postcranial skeletal pneumaticity (PSP) manifests first in the neural arches of the posterior cervical vertebrae, extending anteriorly and/or posteriorly along the vertebral column in more derived forms. The presacral centra of the early‐branching sauropodomorphs examined herein are apneumatic. Unambiguous evidence for PSP cannot be determined solely from external indicators; internal chambers communicate with both undivided fossae and subdivided fossae, but subdivided fossae do not always communicate with internal chambers. Although not always practical, x‐rays or CT/synchrotron scanning are currently the only reliable ways of directly observing the communication of external pneumatic fossae with internal pneumatic chambers. Future research is needed to resolve the exact timing of the spread of PSP along the vertebral column and its invasion into the centra among early‐branching sauropodomorphs. We also urge caution in defining new bone structures in fossil taxa until the characteristics of PSP are better constrained and understood.

PSP appears to show a broad phylogenetic signal, with increased development in later‐branching sauropodomorphs. We find no link between pneumaticity and body size in early‐branching sauropodomorphs. We show that PSP first appears in lagerpetids during the Carnian and in theropods and sauropodomorphs during the Norian. This provides additional support for the idea that PSP evolved independently in three avemetatarsalian lineages.

## AUTHOR CONTRIBUTIONS

SLB conceived, designed and performed the experiments, analysed and interpreted the data, produced figures and tables and led the writing of the manuscript. DS facilitated CT scanning of specimens, analysed and interpreted the data, produced figures, contributed to drafts of the manuscript and approved the final manuscript. PU interpreted the data, contributed to drafts of the manuscript and approved the final manuscript. PMB facilitated CT scanning of specimens, interpreted the data, contributed to drafts of the manuscript and approved the final manuscript. PA conducted CT scanning, analysed the data, contributed to drafts of the manuscript and approved the final manuscript. PDM interpreted the data, made critical revisions of the manuscript and approved the final draft.

## Data Availability

The data that support the findings of this study are available from the corresponding author upon reasonable request.
